# Unravelling neutropenic enterocolitis: insights from gut microbiota, and intestinal barrier analyses

**DOI:** 10.1186/s40164-025-00661-4

**Published:** 2025-05-16

**Authors:** Natacha Kapandji, Maud Salmona, Anaïs Lemoine, Guillaume Ulmann, Julien Calderaro, Brigitte Roche, Nathalie Kapel, Lucie Biard, Etienne Lengline, Jérôme Le Goff, Christophe Rodriguez, Muriel Thomas, Lara Zafrani

**Affiliations:** 1 Intensive Care Unit, Saint Louis Academic Hospital, AP-HP, 1 avenue Claude Vellefaux, Paris, 75010 France; 2 UMR1319, Micalis Institute, INRAe, AgroParisTech, Université Paris-Saclay, Jouy-en-Josas, France; 3 Gut, Liver & Microbiome Research (GLIMMER), FHU, Paris, France; 4 Virology Department, Saint Louis Academic Hospital, AP-HP, Paris, France; 5 Team Insight, INSERM U976, Université Paris-Cité, Paris, France; 6 Clinical Chemistry Department, Cochin Academic Hospital, AP-HP, Paris, France; 7 Pathology Department, Henri Mondor Academic Hospital, AP-HP, Créteil, France; 8 Pathology Department, Saint Louis Academic Hospital, AP-HP, Paris, France; 9 Functional Coprology Laboratory, Pitié Salpêtrière Academic Hospital, AP-HP, Paris, France; 10 Clinical Trial Unit, Saint Louis Academic Hospital, AP-HP, Paris, France; 11 Hematology Department, Saint Louis Academic Hospital, AP-HP, Paris, France; 12 Department of Microbiology, Henri Mondor Academic Hospital, AP-HP, Créteil, France; 13 INSERM UMR 944, Université Paris-Cité, Paris, France

**Keywords:** Neutropenic enterocolitis, Gut microbiota, Interleukin-6 family, Citrulline, Butyrate

## Abstract

**Background:**

Neutropenic enterocolitis (NE) is a severe digestive complication of chemotherapy, primarily affecting patients with acute myeloid leukemia (AML). We hypothesized that NE is linked to intestinal barrier dysfunction and gut dysbiosis.

**Methods:**

Sixty-five AML patients undergoing induction chemotherapy were included in this prospective monocentric cohort. Among them, 26 patients (40%) were diagnosed with NE. Stool samples were subjected to bacterial load quantification (all bacteria quantitative PCR), 16s rRNA metagenomic analysis, and short-chain-fatty-acids quantification. Additionally, fecal calprotectin and human 𝛃-defensin 2 along with plasmatic inflammatory cytokines, and citrulline levels were measured. Human transcriptomic analysis was conducted on samples obtained from anatomical specimens of colectomies of NE patients.

**Results:**

Gut microbiota underwent significant alterations after chemotherapy, transitioning from a diverse and balanced enterotype to enterotypes exhibiting a reduced α-diversity, an increased abundance of *Enterococcus faecalis*, and a decreased abundance of butyrate-producing genera, which correlated with a decreased fecal concentration of butyrate. Simultaneously, post-chemotherapy, plasma citrulline concentrations decreased indicating enterocyte damages. Finally, human transcriptomic analysis found a significant upregulation of the JAK-STAT signaling KEGG pathway in the colons of NE patients encompassing cytokines (IL-6, OSM-OSMR) that play a pivotal role in sustaining local inflammation within the digestive tract.

**Conclusions:**

This work reaffirms the significant influence of chemotherapy on the gut microbiota and the integrity of the enterocyte barrier. Severe NE is marked by the development of a local inflammatory response that may be induced by the reduction in butyrate levels.

**Trial registration:**

The study was registered on Clinicaltrials.gov (identifier: NCT04438278).

**Graphical Abstract:**

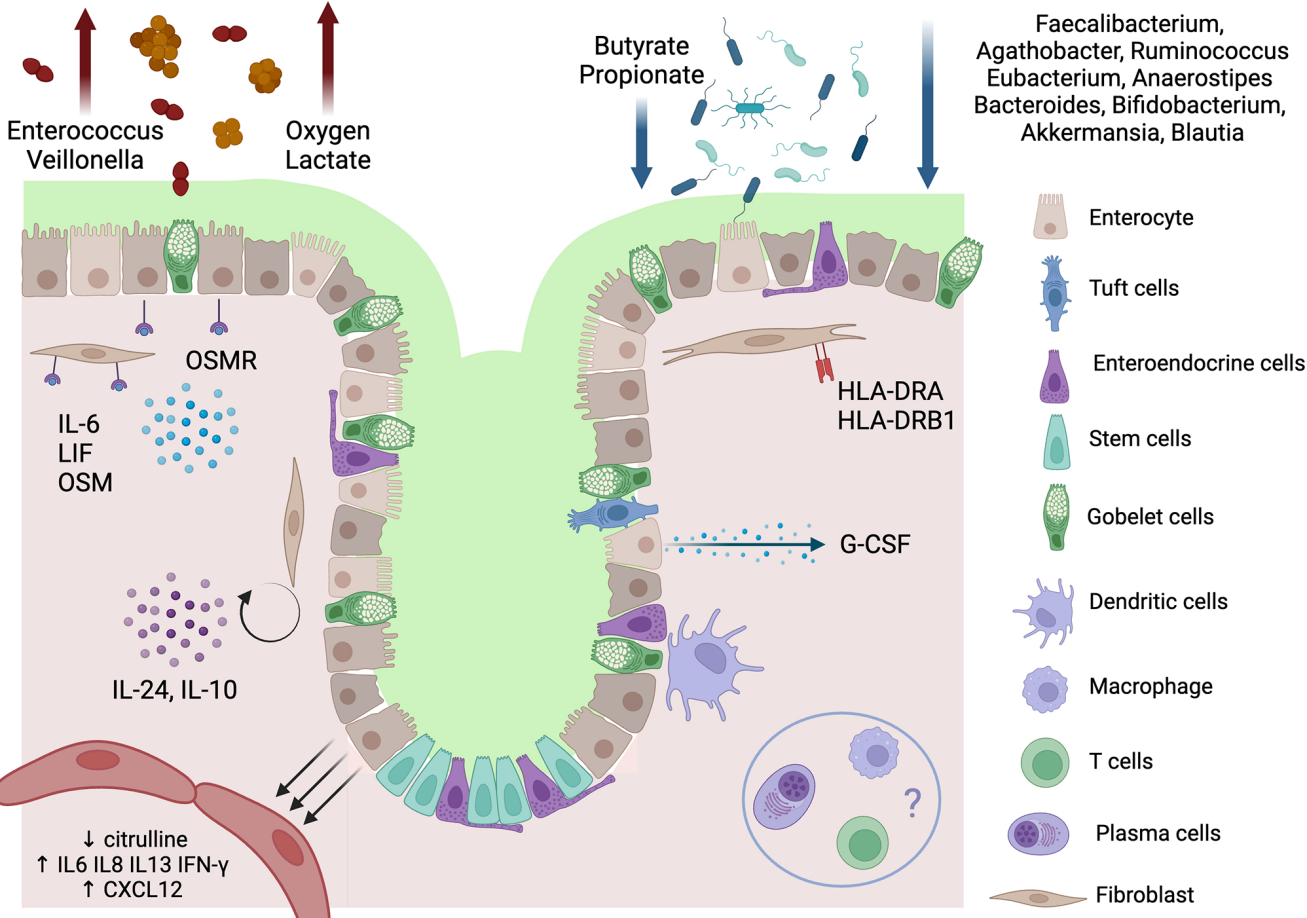

**Supplementary Information:**

The online version contains supplementary material available at 10.1186/s40164-025-00661-4.

## Background

Neutropenic enterocolitis (NE) is a significant digestive complication in patients with chemotherapy-induced neutropenia. It accounts for up to 28% of the acute abdominal syndromes in this population [1]. Acute myeloid leukemia (AML) is the primary condition associated with NE. Its management often necessitates admission to the Intensive Care Unit (ICU) due to septic shock, gastrointestinal bleeding or digestive perforation. In the last studies, NE is associated with high mortality rates of 38.8% [2] and 42% [3]. Diagnosis remains a challenge for clinicians. Nesher and Rolston proposed a set of criteria consisting of major criteria (including neutropenia < 500.10^9^ neutrophils/L, fever > 38.3 °C, and bowel wall thickening on CT-scan or ultrasound > 4 mm in cross-section and > 30 mm longitudinally) and minor criteria (including abdominal pain, distension or cramping, diarrhea, and lower gastrointestinal bleeding) [4]. However, differential diagnosis – foremost among which infectious colitis due to *Clostridium difficile* and *Cytomegalovirus* – must still be carefully ruled out [5].

The pathophysiology underlying NE remains poorly understood. Proposed mechanisms, derived from pathological observations, include local toxicity of chemotherapeutic agents, necrotizing of specific tumoral infiltration, coagulation disorders leading to intramural bleeding, and microbial invasion [2, 5]. In the fields of stem-cell transplantation, recent research endeavors have concentrated on unraveling the intricate connections between the gut bacteriome and chemotherapy-related toxicities [6], and outcomes [7, 8]. These investigations have highlighted various correlations, including those pertaining to infection, graft-versus-host disease, tumorigenesis, and mortality. Thus, we hypothesize that NE’s development could result from a severe disruption of the symbiotic relationship between the gut microbiota and the intestinal barrier.

To explore this hypothesis, we conducted a longitudinal follow-up study of patients receiving their first chemotherapy for AML, focusing on severe patients who developed NE. We examined alterations in gut microbiota’s composition, fecal production of short-chain fatty acids (SCFAs), and evaluated biomarkers of enteral trophicity and systemic inflammation. Additionally, in patients requiring colectomy, we analyzed local genomic expression modifications associated with NE. Through this comprehensive analysis, we aim to gain deeper insights into the underlying mechanisms contributing to the pathogenesis of NE.

## Methods

Detailed methods are provided in Supplemental data.

### Patient recruitment, sample collection and clinical data recording

**Fig. 1:**
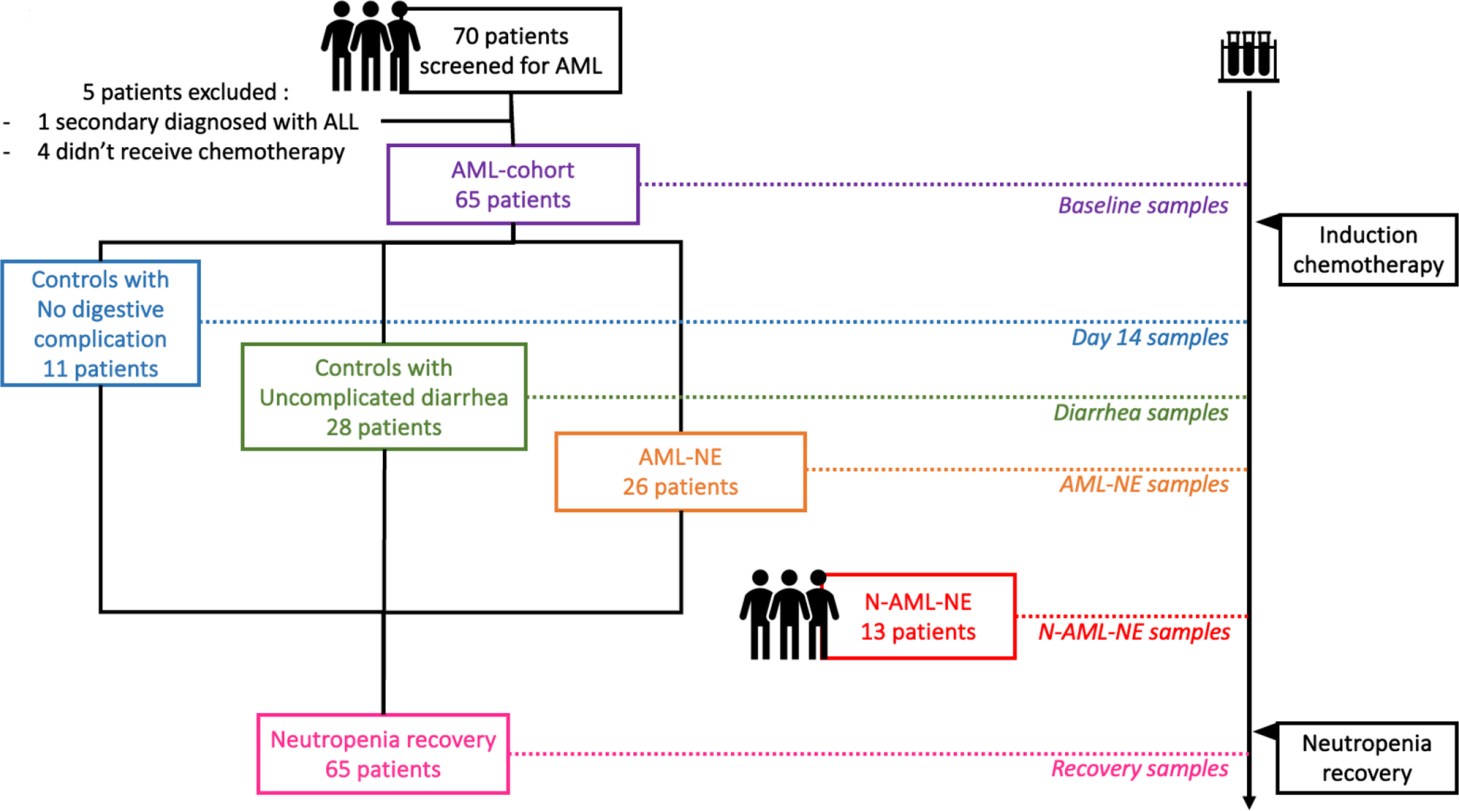
Flow-chart and samples collection. The underlying hematological diseases of the 13 N-AML-NE patients was aggressive lymphomas for 11, multiple myeloma for 1, and acute lymphoblastic Philadelphia-chromosome leukemia for 1

Patients receiving induction chemotherapy for AML between September 2020 and August 2022 were prospectively recruited into this monocentric cohort study. Patients with promyelocytic AML were excluded. They received chemotherapy and broad-spectrum antibiotics in case of neutropenic fever or septic complication at the discretion of the clinician. Demographic (Age, sexe, gender, comorbidities, etc.) and clinical data were prospectively collected. During neutropenia, patients presenting the 3 major criteria defined by Nesher and Rolston [4] and no alternative diagnosis were diagnosed with NE and constituted the AML-NE group. The remaining patients constituted the AML-control group, which was further divided into two subgroups: the AML-control diarrhea (+) group, comprising patients with uncomplicated diarrhea not related to NE, and the AML-control diarrhea (-) group, consisting of asymptomatic patients. Additionally, patients admitted to the ICU for NE with other underlying hematological diseases than AML constituted the N-AML-NE group (Fig. 1). Severe NE was defined as the presence of at least one organ dysfunction, including kidney or liver impairment, hemodynamic instability, or acute respiratory failure requiring ICU admission. Blood and feces samples were collected at baseline (before initiation of chemotherapy), at Day 14 (aligning with the median onset of NE [2]), in case of uncomplicated diarrhea or when NE was diagnosed, and after neutropenia recovery. All samples were stored at -80 °C within 4 h of collection. The study was approved by the ethics committee “Comité de Protection des Personnes Ile de France VII” (N° ID-RCB: 2019-A02172-55) and was registered on Clinicaltrials.gov (identifier: NCT04438278).

### Fecal samples

Bacterial load was evaluated by quantitative 16s ribosomal RNA-coding gene PCR analysis, and bacterial composition was assessed by targeting the V3 and V4 hypervariable regions of the 16 S ribosomal RNA-coding gene employing the primers S-D-Bact-0341-b-S-17 and S-D-Bact-0785-a-A-21 [9] and the Illumina MiSeq platform (Illumina^®^, San Diego, California, USA). Subsequently, concentrations of the different SCFAs (acetate, propionate, butyrate, valerate, isobutyrate, isovalerate, caproate, and isocaproate) were measured using the Gas-chromatograph 7890B (Agilent^®^, Santa-Clara, CA, USA). Fecal calprotectin (f-calprotectin), a protein secreted by neutrophils in the inflamed gut and currently used to monitor inflammatory bowel diseases, was used as a marker of neutrophil activation in the gut. Its levels were measured using a chemiluminescent immunoassay on an automated system (Liaison^®^ XL, DiaSorin). The detection limit was 5 µg/g of feces. Human 𝛽-defensin 2 (f-hBD2) is an antimicrobial peptide secreted by the gastrointestinal epithelium in response to inflammation or infection. It was quantified in the feces by ELISA (Immundiagnostik^®^ AG, Germany) according to manufacturer instructions. Absorbance measurements were performed on a BioTek Epoch2 microplate reader (Agilent^®^) and data analyzed using the BioTek Gen5 software (Agilent^®^). The quantification threshold was 0,2 ng/g of feces.

### Plasma samples

Plasmatic citrulline, commonly used in short bowel syndromes as a marker of enterocyte mass, was used as a biomarker of enteral trophicity. Citrulline concentration was measured by ion-exchange high performance liquid chromatography (Aminotac Amino Acid Analyzer, Jéol^®^, Croissy-sur-Seine, France). Cytokines and chemokines concentrations were determined using Meso Scale Discovery (MSD) electrochemiluminescence method with the MESO QuickPlex SQ120 (MSD^®^, Rockville, Maryland, USA) device and the V-PLEX Proinflammatory Panel 1 (human) kit.

### Transcriptomic analysis

When NE patients required digestive resection, colonic samples were collected and analyzed by the pathology department. Paraffin-embedded fragments were used for metatranscriptomic analysis. Results were compared to samples obtained from anatomical specimens of colectomies performed for colonic tumors, from areas distant from the tumor site.

### Data analysis

The R software with the different specific packages was used for the metagenomic and metatranscriptomic analyses. Statistical analyses were carried out using Jamovi version 2.3.21.0 (RRID: SCR_016142) [10] and GraphPad Prism version 10.1.1 (270) for MacOS GraphPad Software^®^, Boston, Massachusetts USA (RRID: SCR_002798). Missing data were not imputed. All comparisons were bilateral and a p-value < 0.01 was considered statistically significant. Reporting follow the STORMS (Strengthening The Organizing and Reporting of Microbiome Studies) guidelines [11].

### Data availability

All sequence data were deposited in the National Center for Biotechnology Information Sequence Read Archive (Bioproject ID PRJNA1027838).

## Results

Sixty-five patients were included in the AML-cohort. Among them, 45 (69%) presented hyperleukocytosis, 18 (28%) leukostasis, 26 (40%) diffuse intravascular coagulopathy and 23 (35%) tumor lysis syndrome. Forty-three (66%) received hydroxyurea, and 39 (60%) dexamethasone before induction chemotherapy. Thirty-eight patients (59%) received their induction chemotherapy in the ICU. Antibiotics were prescribed before induction chemotherapy in 44 patients (68%)(Table 1).

**Table 1:** Clinical characteristics of the AML-cohort

			AML-cohort (*n* = 65)		*p*
			AML-Controls (*n* = 39)	AML-NE (*n* = 26)	
Age (years)			54 ± 14		
			55 ± 13	52 ± 13	0.40
Male – n (%)			42 (65)		
			23 (59)	19 (73)	0.24
AML group according to the WHO’s classification – n (%)					
AML with recurrent genetic abnormalities			32 (82)	19 (73)	
AML with myelodysplasia-related changes			3 (8)	0	0.14
AML, not otherwise specified			4 (10)	7 (27)	
**Patients’ characteristics at admission**					
AML complications – n (%)		Hyperleukocytosis	45 (69)		
			24 (64)	20 (77)	0.27
		Leukostasis	18 (28)		
			11 (28)	7 (27)	0.91
		DIC	26 (40)		
			16 (41)	10 (39)	0.84
		Tumor lysis syndrome	23 (35)		
			17 (44)	6 (23)	0.09
SOFA score – n (%)			3 [1–4]		
			3 [1–4]	3 [2–4]	0.63
ICU admission – n (%)			38 (59)		
			18 (46)	20 (77)	0.01
SAPS II in ICU patients [IQR]			29 [23–35]		
			32 [26–41]	28 [23–33]	0.20
Malnutrition – n (%)			6 (9)		
			2 (5)	4 (15)	0.21
Digestive disorders * – n (%)			10 (15)		
			4 (10)	6 (23)	0.18
Antibiotic treatment before chemotherapy – n (%)			44 (68)		
			25 (64)	19 (73)	0.45
Duration of antibiotics (days) [IQR]			5 [4–7]		
			6 [4–7]	5 [2–7]	0.11
Monotherapy – n (%)			33 (75)		
			19 (76)	14 (74)	0.02
Antibiotic with anti-anaerobic activity – n (%)			22 (50)		
			12 (28)	10 (53)	0.86
Piperacillin-Tazobactam or Carbapenem – n (%)			10 (23)		
			10 (40)	0	0.001
**Received treatments from AML**					
Aracytine and Anthracycline combination – n (%)			60 (92)		
			34 (87)	26 (100)	0.08
Anti-CD33 treatments – n (%)			9 (14)		
			4 (10)	5 (19)	0.47
FLT3-inhibitor treatments – n (%)			15 (23)		
			7 (18)	8 (31)	0.23
Hydroxyurea – n (%)			43 (66)		
			24 (62)	19 (73)	0.34
Dexamethasone – n (%)			39 (60)		
			20 (51)	19 (73)	0.08
**Infectious complications during neutropenia**					
Febrile neutropenia – n (%)			52 (80)		
			34 (87)	18 (69)	0.11
Oral mucositis – n (%)			33 (51)		
			13 (33)	20 (77)	< 0.001
Herpes Simplex Virus recurrences – n (%)			17 (26)		
			4 (10)	13 (50)	< 0.001
Clinically defined infectious complications** – n (%)			11 (17)		
			5 (13)	6 (23)	0.61
Pulmonary invasive fungal infections – n (%)			5 (8)		
			3 (8)	2 (8)	1.00
Bloodstream infections – n			26		
			11	15	0.04
Multiple pathogens			2	5	0.66
Related to catheter colonization			6	2	0.04
*Enterococcus spp.*			1	4	0.36
*Enterobacteriaceae*			0	7	0.01
*Candida spp.*			1	2	1.00
**Antibiotics administered during neutropenia**					
Number of days with antibiotic post chemotherapy [IQR]			23 [19–30]		
			24 [19–30]	23 [20–29]	0.88
Azole– n (%) *Days [IQR]*			16 (25)		
			9 (23)	7 (27)	0.72
			7 [4–14]		
			7 [4–14]	7 [6–13]	0.83
Cephalosporin *–* n (%) *Days [IQR]*			43 (66)		
			25 (64)	18 (69)	0.67
			10 [7–15]		
			11 [7–18]	10 [7–15]	0.31
Penicillin *–* n (%) *Days [IQR]*			46 (71)		
			31 (80)	15 (58)	0.06
			10 [5–19]		
			10 [6–20]	9 [5–18]	0.54
Carbapenem – n (%) *Days [IQR]*			26 (40)		
			21 (54)	5 (19)	0.005
			8 [5–13]		
			8 [5–13]	8 [4–11]	0.87
Vancomycin – n (%) *Days [IQR]*			23 (35)		
			15 (39)	8 (31)	0.53
			10 [7–13]		
			10 [6–12]	10 [7–14]	0.70
Aminoside – n (%)			7 (11)		
			4 (10)	3 (12)	1.0
**Outcomes**					
Duration of neutropenia (days) [IQR]			21 [20–24]		
			21 [20–24]	21 [20–22]	0.48
Hospital length of stay (days) [IQR]			31 [28–38]		
			30 [28–36]	33 [29–46]	0.04
Remission rate at discharge – n (%)			52 (80)		
			31 (80)	21 (81)	0.90
In-hospital mortality rate – n (%)			12 (19)		
			7 (18)	5 (19)	1.00
Malnutrition at discharge – n (%)			27 (42)		
			13 (33)	14 (54)	0.10
1-year remission rate – n (%)			36 (72)		
			22 (71)	14 (74)	1.00
1-year mortality rate – n (%)			20 (31)		
			11 (28)	9 (36)	0.51

* Digestive disorders upon admission include diarrhea, vomiting and abdominal pain

** Clinically defined infectious complications were highly suspected infections with no bacteriological confirmation. They included: 3 skin infections, 5 pneumonias, and 3 Ear-Nose-and-Throat infections

AML: Acute myeloid Leukemia; DIC: Disseminated intravascular coagulation; NE: Neutropenic Enterocolitis; SAPS II: Simplified Acute Physiology Score; SOFA score: Sequential Organ Failure Assessment; WHO: World Health Organization

Following induction chemotherapy, the 65 patients experienced neutropenia for a median of 21 [20–24] days. Twenty-six (40%) were diagnosed with NE (AML-NE). The remaining 39 patients (60%) constituted the AML-control group. Among them, 28 (43%) experienced uncomplicated diarrhea (AML-C diarrhea (+)), while 11 patients (17%) did not have any digestive complications (AML-C diarrhea (-)) (Fig. 1). The baseline characteristics of the AML-NE patients did not differ from the rest of the cohort, and they did not receive more antibiotics prior to induction chemotherapy (Table 1 and Supplemental Figure S1).

During neutropenia, AML-NE patients exhibited a higher incidence of bloodstream infections (15 vs. 11; *p* = 0.04) with a notable prevalence of *Enterobacteriaceae* (Supplemental Table S1). They also presented more oral mucositis [20 (77%) vs. 13 (33%); *p* < 0.001], and Herpes Simplex virus (type 1 and 2) recurrences [13 (50%) vs. 4 (10%); *p* < 0.001]. However, antibiotic treatments did not differ in either antibiotic class or duration (Table 1). No significant differences were observed in 1-year outcomes (Supplemental Figure S2).

Additionally, 13 patients admitted to the ICU for NE with other underlying hematological diseases, had samples collected and formed the N-AML-NE group. Most of these patients (8/13) had undergone autologous stem-cell transplantation. They were not clinically different from the AML-NE group (Supplemental Table S2).

### Bacterial load and 𝛼-diversity significantly decreased after chemotherapy in all patients

**Fig. 2:**
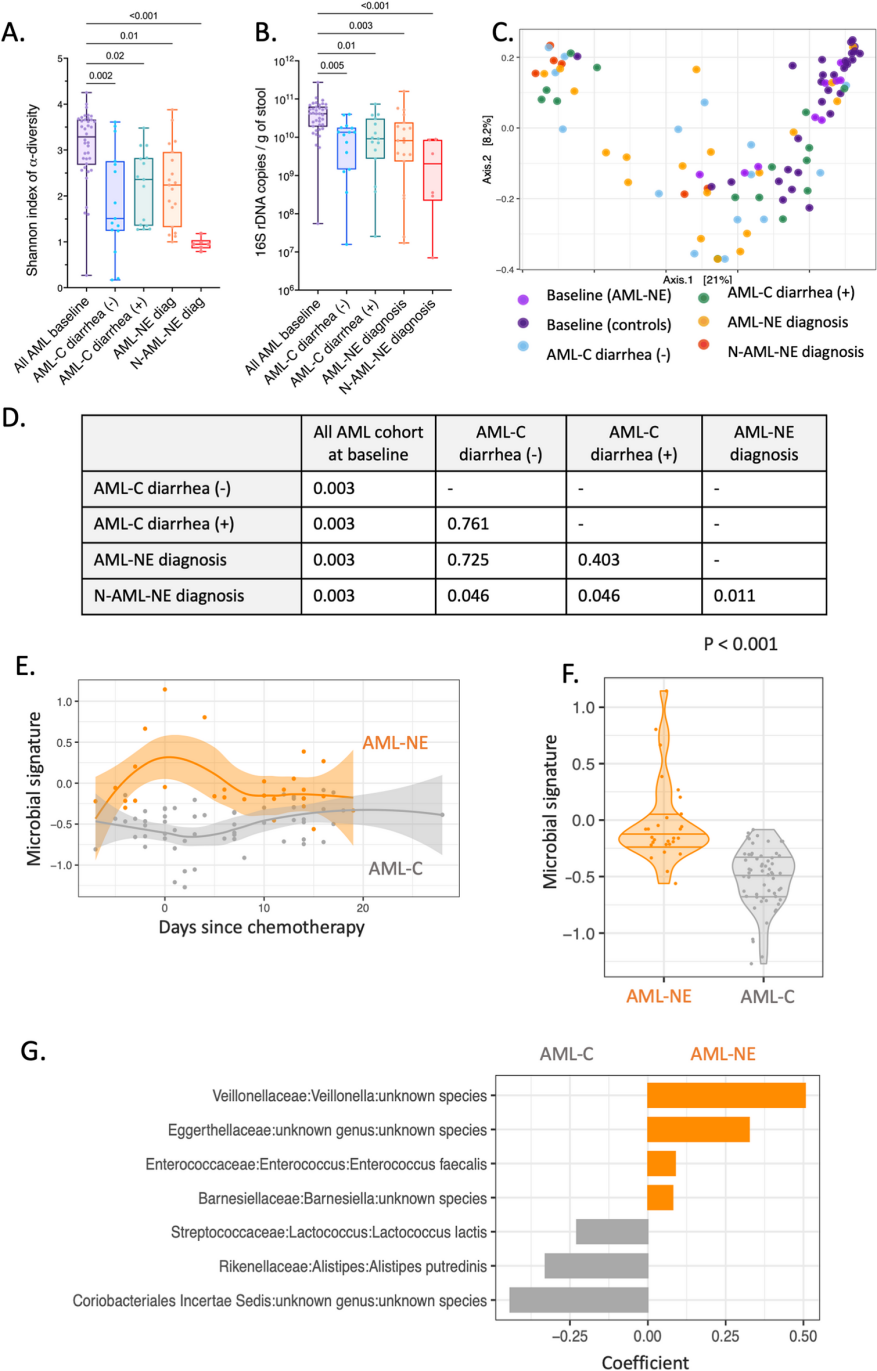
Targeted 16s rRNA metagenomic analysis of gut microbiome (α and β-diversity). Box plots presenting levels of (**A**) Shannon α -diversity index and (**B**) bacterial load (expressed as number of bacteria / g of feces) between the 4 different clinical timepoints: before chemotherapy in all AML patients (*n* = 38), at day 14 for AML-controls diarrhea (-) (*n* = 15), at diagnosis of diarrhea for AML-controls diarrhea (+) (*n* = 15), at diagnosis of NE for AML-NE (*n* = 19), and N-AML-NE (*n* = 6). *p-values* were calculated using a non-parametric two-sided Kruskal-Wallis test with Dunn’s multiple comparisons tests. A *p-value* < 0.01 was considered statistically significant. (**C**) Dot plot illustrating the coordinates of the Bray-Curtis matrix distances of each sample on Principal Coordinates Analysis (PCoA). Dots were colored based on their associated clinical timepoint. (**D**) Permutated Multivariate Analysis of Variance (PERMANOVA) analysis of Bray-Curtis distances. A *p-value* < 0.001 was considered statistically significant. (**E**) Violin plot and (**F**) regression curve with 95%CI comparing the dynamic modifications in the AML-NE group (*n* = 26) to the rest of the AML controls (*n* = 39). (**G**) Bar plot describing taxa contributing to the dynamic microbial signature of NE

The taxonomic identification generated an average of 92,038 reads per sample, and a total of 439 operational taxonomic units (OTUs) were annotated. Regarding α-diversity, all indexes (number of identified OTUs, Shannon and Simpson indexes) were lower in the post-chemotherapy samples (*p* < 0.001), with Shannon index decreasing from 3.3 [2.7–3.7] before chemotherapy to 2.2 [1.3–3.0] in the AML-NE group at NE’s diagnosis (Fig. 2.A). Similarly, bacterial load was 4.1 10^10^ [1.9 10^10^; 6.4 10^10^] bacteria / gramme of feces in the AML-cohort before chemotherapy and decreased in all groups (*p* < 0.001) down to 9.2 10^9^ [2.3 10^9^; 2.5 10^10^] in the AML-NE group at NE’s diagnosis (Fig. 2.B). Although the N-AML-NE group had the lowest Shannon index and bacterial load, there was no statistical difference between the 2 NE groups (AML and N-AML) and the 2 post-chemotherapy AML control groups [diarrhea (+) and diarrhea (-)]. Regarding the impact of antibiotics, the initiation of antibiotics prior to chemotherapy was not significantly associated with a reduction in bacterial load (*p* = 0.26) or α-diversity indices in baseline samples (Supplemental Figure S3.A). Moreover, neither the use of broad-spectrum antibiotics (piperacillin-tazobactam or carbapenems), nor prolonged antibiotic treatments was associated with a significant reduction in α-diversity.

### Dynamic taxonomic analysis identified a microbial signature associated with NE

When Bray-Curtis distances were subject to PERMANOVA analysis, baseline samples from the AML-cohort taken before chemotherapy exhibited distinct clustering from all post-chemotherapy samples (*p* = 0.003). After chemotherapy, all 4 groups were not statistically different (Fig. 2.C and D). However, the dynamic analysis of the AML-cohort identified a microbial signature associated with AML-NE’s patients (*p* < 0.001) (Fig. 2.E and F). This signature was positively characterized in sequence by phylotypes related to *Veillonella*, *Eggerthellaceae* family, *Enterococcus faecalis*, and *Barnesiella*. In contrast, phylotypes associated with AML-controls included those from the *Coriobacteriaceae Incertae Sedis* family, *Alistipes putredinis*, and *Lactococcus lactis* (Fig. 2.G).

### Unsupervised analysis identified two enterotypes associated with NE

**Fig. 3:**
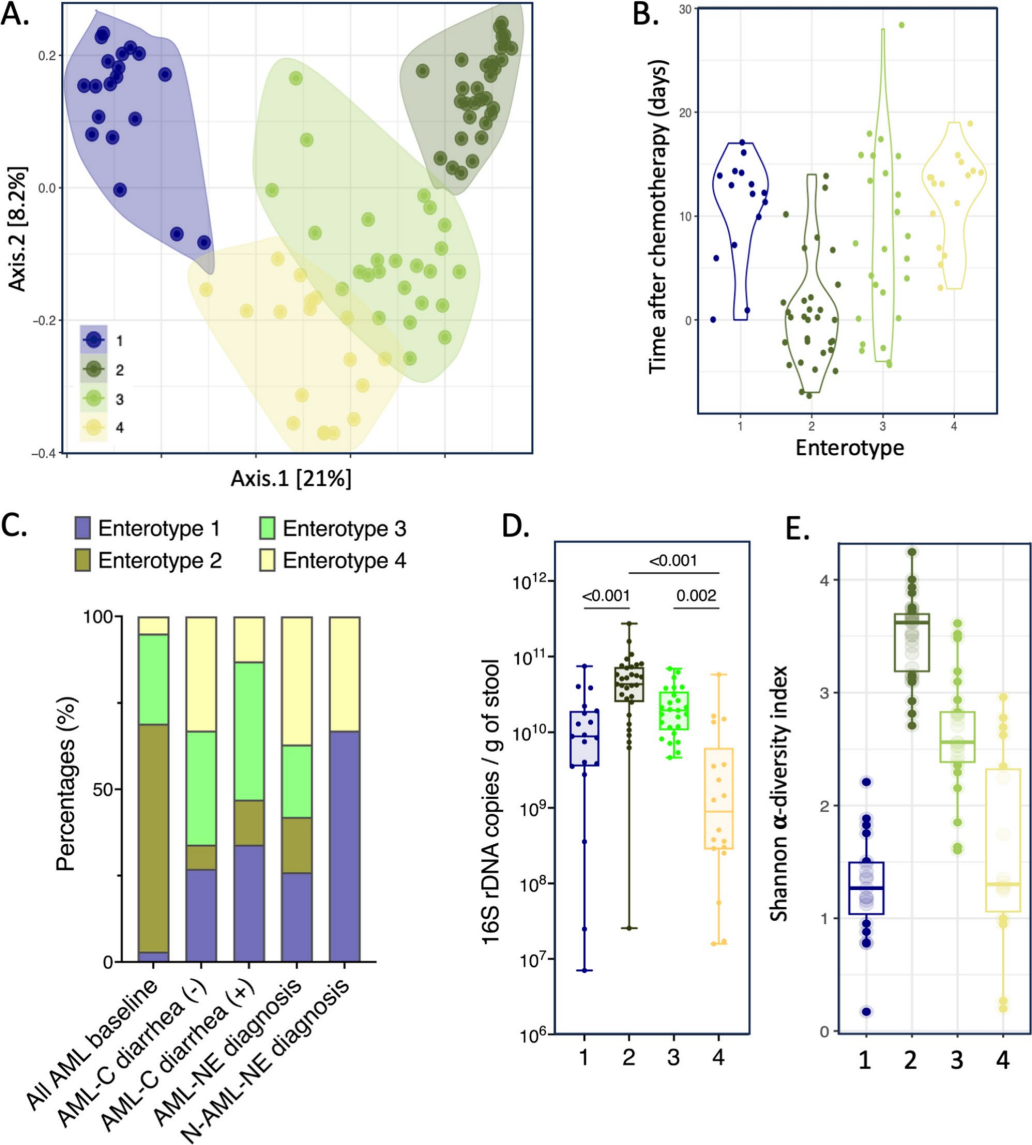
Unsupervised analysis of 16s rRNA data identified 4 distinct enterotypes. (**A**) Dot plot depicting coordinates of each sample on the principal co-ordinate analysis (PCoA) performed using the Bray-Curtis matrix distance. Samples were clustered using hierarchical k-means, with each cluster representing an enterotype. (**B**) Comparison of the time elapsed from the initiation of chemotherapy in the AML-cohort between the enterotypes. *p-value* was computed with bilateral Wilcoxon test. (**C**) Bar plot showing the distribution of the 4 enterotypes among the different clinical timepoints (All AML baseline, AML-C diarrhea (-) at Day 14, AML-C diarrhea (+), AML-NE, and N-AML-NE at diagnosis). Box plots depicting the comparisons of (**D**) bacterial load (bacteria / g of feces), and (**E**) Shannon α -diversity index between the 4 enterotypes [enterotype 1 (*n* = 19), enterotype 2 (*n* = 31), enterotype 3 (*n* = 25) and enterotype 4 (*n* = 18)]. Two tailed *p-values* were performed using Kruskal-Wallis test with Dunn’s multiple comparisons tests. *p-value* < 0.01 was considered statistically significant

Unsupervised analysis identified 4 clusters named enterotypes (Fig. 3.A), with a temporal switch from a predominance of an enterotype 2 to a predominance of enterotypes 1 and 4 (Fig. 3.B). These enterotypes were associated with clinical time-points (*p* < 0.001). Indeed, 66% (25/38) of samples taken before chemotherapy presented the enterotype 2 and 72% (18/25) of samples taken at the diagnosis of NE (AML or N-AML) presented the enterotype 1 or 4 (Fig. 3.C). This distribution of enterotypes remained stable both at baseline (*p* = 0.08) (Supplemental Figure S3.B) and during neutropenia regardless of antibiotic class and duration.

Regarding microbial characteristics, fecal bacterial load and α-diversity also differed significantly among the enterotypes (*p* < 0001) (Fig. 3.D and E). Enterotype 2 was the richest with a bacterial load of 4.3 10^10^ [2.5 10^10^– 7.3 10^10^]. It also exhibited the highest Shannon α-diversity index (3.6 [3.2–3.7]). It was characterized by high proportions of members of the *Eubacteriales* order: *Agathobacter*,* Anaerostipes*,* Blautia*,* Dorea*, *Roseburia*, but also *Butyricicoccus*, *Ruminococcus* and members of the *Eubacterium siraeum* group. It also included *Akkermansia*, *Bifidobacterium* and *Collinsella*. Enterotypes 1 and 4 had the lowest bacterial loads (8.8 10^9^ [3.5 10^9^– 1.9 10^10^] and 8.9 10^9^ [2.8 10^9^– 6.2 10^9^] respectively) and the lowest Shannon α-diversity indexes (1.3 [1.0–1.5] and 1.3 [1.0–2.4] respectively). Enterotype 1 was characterized by a strong predominance of *Enterococcus*.

### Enterotypes associated with NE were characterized by a depletion in short-chain-fatty acids producing genera and low SCFA fecal concentrations

**Fig. 4:**
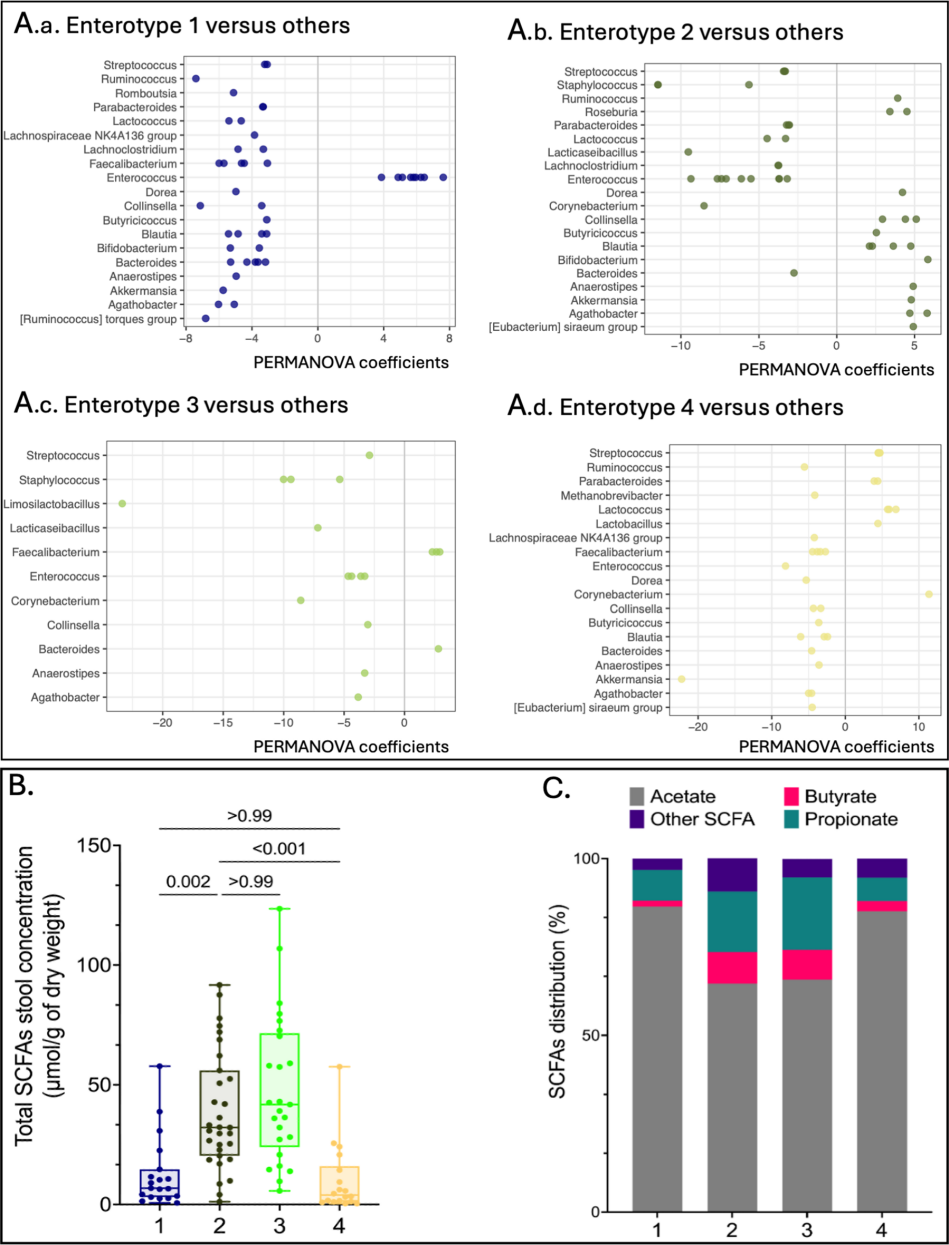
Enterotypes 1 and 4 demonstrated a reduced production of fecal short-chain fatty acids. (**A**) Bar plots presenting for each enterotype (A.a to A.d) the differential abundance of the significant genera (adjusted p-value < 0.01). (**B**) Box plots comparing the total SCFAs fecal concentrations expressed in µmol / g of dry feces weight among the 4 enterotypes [enterotype 1 (*n* = 19), enterotype 2 (*n* = 31), enterotype 3 (*n* = 25) and enterotype 4 (*n* = 18)]. (**C**) Bar plot showing the distribution in percentages of the majority SCFAs namely acetate, butyrate, and propionate for each enterotype. “Other SCFAs” gathered valerate, isovalerate, isobutyrate, caproate, and isocaproate

Enterotype 1 and 4 exhibited a lower representation of bacterial genus that were predominant in enterotype 2, and which are known to be SCFAs-producing genera: *Anaerostipes*,* Agathobacter*, *Akkermansia*,* Blautia*,* Bifidobacterium*,* Bacteroides*,* Butyricicoccus*,* Eubacterium*,* Faecalibacterium*, and *Ruminococcus* (Fig. 4.A.a to A.d). Consistently, enterotypes 1 and 4 had significantly lower total SCFAs fecal concentrations compared to enterotype 2 with 6.8 µmol/g [3.1–14.7] (*p* = 0.002) and 4.0 µmol/g [1.2–16.0] (*p* < 0.001) respectively, vs. 32.2 µmol/g of dry weight [20.4–56.0] (Fig. 4.B). The decrease was more pronounced for butyrate, representing 1.7% ± 2.6 and 2.9% ± 3.7 of all SCFAs in enterotypes 1 and 4, respectively, vs. 9.0% ± 4.6 in enterotype 2 (*p* < 0.001) (Fig. 4.C). The concentrations of butyrate and propionate were correlated respectively to the number of OTUs of butyrate-producing genera (*R* = 0.57 [0.38–0.71]; *p* < 0.001) (Supplemental Figure S4.A; Supplemental Table S4) and propionate-producing genera (*R* = 0.52 [0.32–0.67]; *p* < 0.001) (Supplemental Figure S4.B; Supplemental Table S4).

### Enterotypes associated with NE exhibited a significant pro-inflammatory cytokine profile

**Fig. 5:**
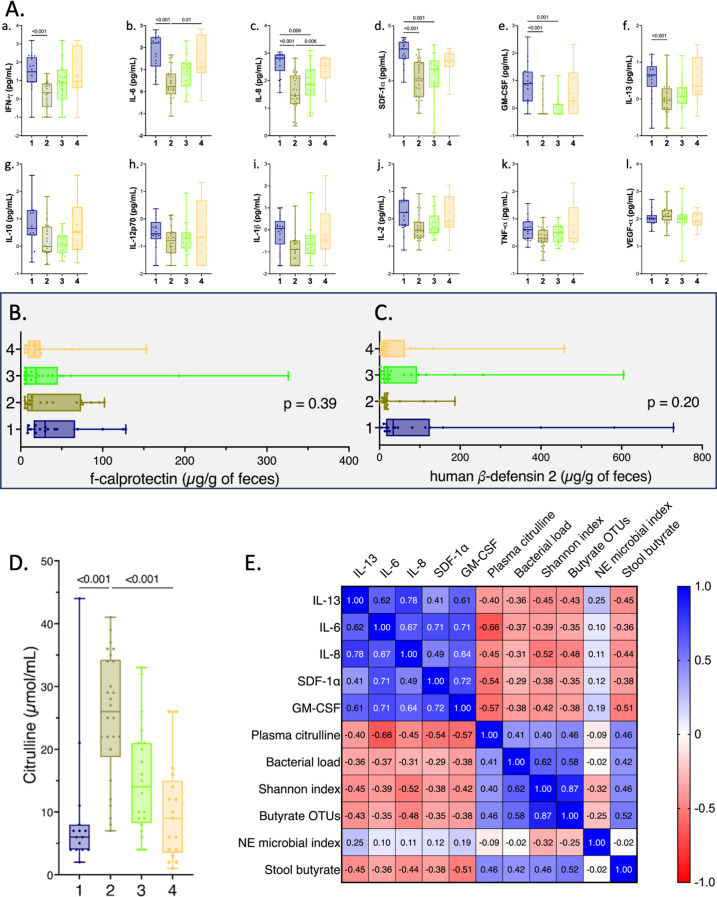
Enterotype 1 and 4 exhibited decreased enteral mass and significant systemic inflammation. (**A**) Box plots comparing the plasmatic concentrations of the following cytokines and chemokines among the 4 enterotypes: IFN- 𝛾 (a), IL-6 (b), IL-8 (c), SDF-1 (d), GM-CSF (4), IL-13 (f), IL-10 (g), IL-12p70 (h), IL-1 β (i), IL-2 (j), TNF-α (k), VEGF (l). Cytokine concentrations were expressed on a base-10 logarithmic scale (pg/mL). (**B**) Box plots comparing f-calprotectin among the 4 enterotypes [enterotype 1 (*n* = 18), enterotype 2 (*n* = 19), enterotype 3 (*n* = 20) and enterotype 4 (*n* = 16)]. (**C**) Box plots comparing the fecal concentration of human β-defensin 2 among the 4 enterotypes [enterotype 1 (*n* = 19), enterotype 2 (*n* = 19), enterotype 3 (*n* = 20) and enterotype 4 (*n* = 16)]. (**D**) Box plots presenting levels of plasma citrulline among the 4 enterotypes [enterotype 1 (*n* = 15), enterotype 2 (*n* = 26), enterotype 3 (*n* = 20) and enterotype 4 (*n* = 17)]. *p-values* were calculated using a non-parametric two-sided Kruskal-Wallis test with Dunn’s multiple comparisons tests. (**E**) Heatmap presenting the matrix of Spearman’s correlation coefficients between IL-6, IL-8, SDF-1, GM-CSF, IL-13, plasma citrulline, bacterial load, Shannon α-diversity index, the number of observed OTUs of butyrate producers, the NE’s bacterial signature coefficient, and the fecal concentrations of butyrate. *p-value* < 0.01 was considered statistically significant

Regarding the panel of 12 plasmatic cytokines tested, significant differences were observed between the 4 enterotypes (*p* < 0.001) for IFN-𝛾, IL-6, IL-8, CXCL12, GM-CSF and IL-13. Compared to enterotype 2, enterotype 1 and 4 had significantly higher levels of circulating IL-6 [162.1 pg/mL [14.0–298.3] (*n* = 17) and 13.2 pg/mL [7.0–390.2] (*n* = 13) respectively, vs. 1.7 pg/mL [0.8–6.6] (*n* = 28)] and IL-8 [654.0 pg/mL [225.1–874.0] (*n* = 17) and 358.6 pg/mL [125.1–654.0] (*n* = 13) respectively, vs. 29.4 pg/mL [14.2–154.7] (*n* = 28)] (Fig. 5.A.a to A.l). However, both f-calprotectin or f-hBD2 levels remained low and showed no significant differences across the 4 enterotypes (*p* = 0.39 and *p* = 0.20), with a median of 18.4 µmol/g of feces [10.3–79.5] for f-calprotectin and 18.6 µmol/g of feces [9.6–49.8] for f-hBD2 (Fig. 5.B and C).

### Enterotypes associated with NE exhibited a significant decrease in plasma citrulline levels which correlated with IL-6 inflammatory profile and low fecal butyrate concentration

Patients classified under enterotypes 1 and 4 exhibited lower plasma citrulline levels (6 µmol/mL [4–8] and 9 µmol/mL [4–15], respectively) compared to enterotype 2 (26 µmol/mL [19–34]) (*p* < 0.001) indicating a severe reduction of enterocyte mass in NE patients **(**Fig. 5.D). Interestingly, a strong correlation was found between plasma citrulline levels and plasma IL-6 levels (*r* = -0.66), as well as with the number of OTUs of butyrate-producing genera (*r* = 0.46) and fecal butyrate concentration (*r* = 0.46) (Fig. 5.E).

### Histopathological and transcriptomic analyses supported the presence of a local immune response in severe NE

**Fig. 6:**
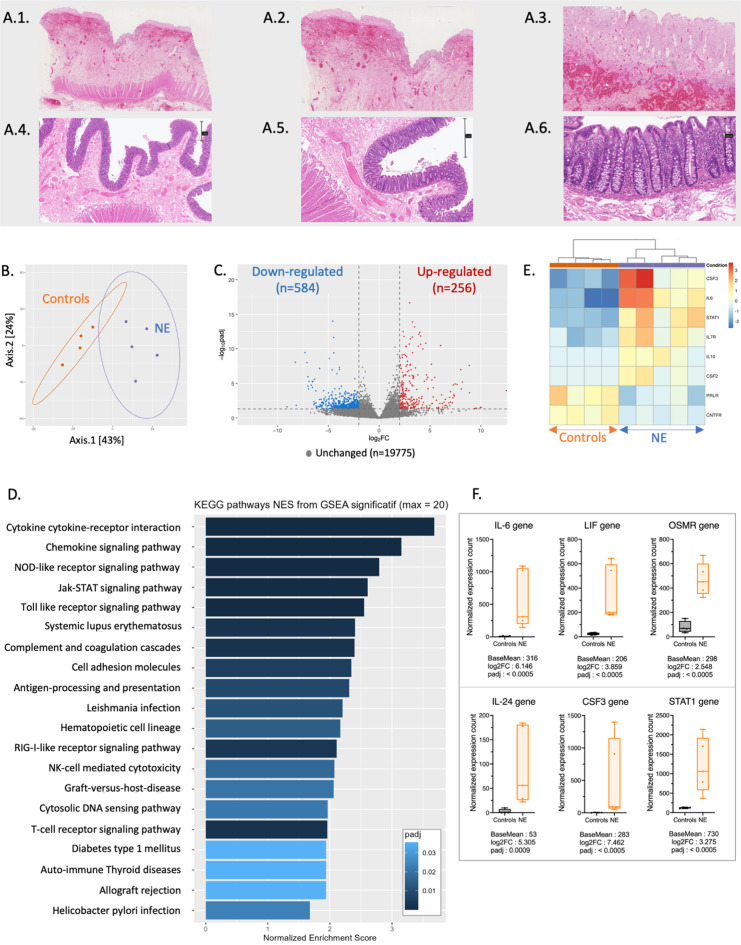
Human transcriptomic and histopathological analysis. (**A**) Histopathological observations of NE specimen (A1 to A3) and control (A4 to A6) using Hematoxylin and eosin stain with X1, X2, and X10 scanning magnifications. Observations A1 to A3 reveal ulcerated mucosa with fibrino-leukocytic exudate, edema, and vascular congestion. Scales are reported on the right of the control images (**B**) Dot plot illustrating the principal transcriptomic coordinates of the 5 NE samples and 4 controls. (**C**) Volcano plot illustrating the down-regulated and up-regulated genes in NE colic samples compared to the controls. Genes were considered unchanged when padj was > 0.05 and L_2_FC < ⎟2⎜. (**D**) Bar plot of the normalized enrichment scores of the 20 most significative up-regulated KEGG-pathways in the NE’s human transcriptomic analysis compared to the controls. (**E**) Heatmap of the Jak-STAT signaling KEGG pathway (hsa04630). (**F**) Bow plots comparing between NE samples and controls the normalized expression count for the most significant genes of the Jak-STAT pathway: STAT1, IL-24, CSF3, and genes from the IL-6 family (IL-6, LIF, OSMR). Specific Mann-Whitney comparison of *padj* < 0.001 was considered statistically significant

Among the 39 patients with NE, 10 particularly severe underwent surgical management, and 6 of them had a colonic resection (Supplemental Table S3). Human transcriptomic analysis compared the 6 colonic samples to 5 controls. The histopathological analysis of the specimens with NE revealed an ulcerated mucosa covered with a fibrino-leukocytic exudate. The submucosa appeared edematous and congested, accompanied by hemorrhagic suffusion (Fig. 6.A). One sample was excluded in each group because the number of identified genes was below 10 000. Principal Component Analysis of global transcriptomic data differentiated between NEs and controls, indicating significant differences in gene expression (Fig. 6.B). The 840 significantly differentially expressed genes defined by a Log_2_Fold Change >⎟2⎜and a p adjusted value < 0.05 (Fig. 6.C) were used to perform unsupervised ranked ontology analysis within the Kyoto Encyclopedia of Genes and Genomes (KEGG) pathways (Fig. 6.D). Among the 185 KEGG pathways, the analysis identified 22 significantly up-regulated ones. The most significative was the JAK-STAT signaling pathway (normalized enrichment score (NES) 2.61, false-discovery rate (FDR) 0.0006, KEGG Pathway hsa04630) that includes the signal transducer and activator of transcription 1 (STAT1), cytokines and receptors from the IL-6 family [IL-6, leukemia inhibitor factor (LIF), oncostatin M (OSM), oncostatin M receptor (OSMR)], cytokines from the IL-10 family (IL-10, IL-24), IL-7 receptor (IL-7R) and growth factors (colony stimulating factor, CSF3) (Fig. 6.E and 6.F). Moreover, among the 22 up-regulated pathways 12 shared up-regulated genes of class II major histocompatibility complex [HLA-DQB1 (NES 8.07, FDR 0.00006), HLA-DRB1 (NES 2.82, FDR 0.008), HLA-DRA (NES 2.04, FDR 0.000008)] and class I major histocompatibility complex [HLA-B (NES 2.34, FDR 0.00006)] (Supplemental Figure S5).

## Discussion

In this cross-sectional study, we investigate changes in the gut microbiota and its production of SCFAs, and we uncovered evidence of local immune activation and inflammatory response despite profound leukopenia. These discoveries offer unprecedented insights into the mechanisms underlying NE.

First, our analysis confirms a pronounced post-chemotherapy dysbiosis in all patients, marked by a significant reduction in bacterial load and α-diversity aligning with previous studies [7, 12, 13, 14]. Indeed, a dysbiotic gut microbiota with low α-diversity has been linked to various post-chemotherapy complications, such as infectious complications [6], neutropenic fever [14], and gastrointestinal mucositis [15]. However, in our cohort, no statistical difference was observed between fecal samples of patients at the diagnosis of NE and post chemotherapy controls. Nonetheless, the significant reduction in α-diversity post-chemotherapy might make it challenging to detect subtle difference among groups. Consequently, our study may be underpowered to identify these small differences. Nevertheless, our results support a continuum of gut dysbiosis after chemotherapy, extending from neutropenic patients without digestive symptoms to those with non-severe symptoms (such as uncomplicated diarrhea or oral mucositis), and further to NE patients. This continuum is also reflected in variations in plasma citrulline levels. Citrulline levels are recognized indicators of active enterocyte mass in various conditions involving acute or chronic enterocyte damages [16].

Interestingly, we were able to identify a microbiological signature associated with NE. Indeed, the dynamic analyses of the gut microbiota revealed an increased proportion of *Enterococcus faecalis and Veillonella* genera among NE patients. *Enterococci* are ubiquitous Gram-positive species that are not predominant in the gut microbiota of healthy individuals. The predominance of *Enterococci* in the gut microbiota has been reported in patients with hematological diseases [7], and in critically ill patients admitted to the ICU [17]. It has been linked to an increased mortality and a higher risk of all-cause infections [18]. *Enterococci* are highly resilient species, capable of tolerating degraded anaerobic conditions, exhibiting both intrinsic and acquired antibiotic resistance, and demonstrating the capacity to form biofilms. Hence, we hypothesize that the observed predominance of *Enterococci* may not directly contribute to the pathophysiology of NE but instead reflects the gut microbiota’s adaptation to environmental changes, such as nutrient deprivation, prolonged antibiotic exposure, and altered aerobic conditions. Similarly, *Veillonella* are Gram-negative, obligate anaerobes found almost exclusively in the human oral cavity. They derive their energy through the fermentation of lactate produced by lactic acid bacteria [19]. The ectopic colonization of the digestive tract by *Veillonella* has been documented in case of inflammatory bowel diseases (IBD) [20] and irinotecan-induced gut toxicity [21]. A recent study hypothesized that the strain’s ability to shift from fermentation to anaerobic respiration using nitrate – more abundant during inflammation – enables its survival in such an environment. This shift in metabolic strategy may be the reason for the increase in its relative proportion [22]. Therefore, the predominance of these genera in NE patients could indicate the gut microbiota’s adaptation to local inflammation, suggested by cytokine levels and the histopathological and transcriptomic analyses of colonic samples.

Indeed, despite profound leukopenia and a deficit of circulating immune cells, NE patients exhibited a high level of circulating proinflammatory cytokines, particularly IL-6. However unlike in active IBD this was not associated with elevated fecal calprotectin [23], suggesting that neutrophils are not the primary mediators in this inflammation. Moreover, the absence of f-BD2 elevation, despite high IL-6 levels, indicates that the gastrointestinal epithelium’s immune response is not actively contributing to the inflammatory process. Instead, this finding, along with the inverse correlation between IL-6 and plasma citrulline levels, suggests that the inflammation is associated with severe reduction of enterocyte mass rather than direct epithelial activation. The analysis of severe NE cases requiring colic resection revealed the activation of the STAT1 transcription activator pathway involving cytokines from the IL-6 family (IL-6, LIF, and OSM-OSMR) [24], the IL-10 family (IL-10, IL-24), as well as the IL-7R. Interestingly, high expression of OSM [25], IL-24 [26], and IL-7R [27] genes have also been reported in colonic samples of patients with IBD. Cytokines from the IL-10 family such as IL-24 are mainly actors of the anti-inflammatory response and can be secreted by T helper 2 cells (Th2), but also by epithelial cells [28] or stromal cells [29]. Stromal cells are non-hematopoietic, non-epithelial, non-endothelial cells including in the gut fibroblasts, myofibroblasts, smooth muscle cells, pericytes, and mesenchymal stromal cells [30]. In IBD, IL-24 produced by colonic subepithelial myofibroblasts induces membrane-bound mucin expression in colonic epithelial cells [29] and contributes to tissue remodeling [26]. Therefore, given the severity of enterocyte damage observed in NE patients, it’s likely that a similar adaptive mechanism exists. On the other hand, cytokines from the IL-6-family play a pivotal role in sustaining local inflammation within the digestive tract. Stromal cells both produce and respond to IL-6 family cytokines, playing a role in regulating the intensity, nature, and duration of immune responses in various tissues [31]. In IBD, the stromal OSM-OSMR axis has been suggested to play a crucial role in the propagation of inflammation [32] and high expression of the OSM gene [25] or elevated plasma OSM levels [33] have been found to predict corticosteroid-dependency [34] and resistance to anti-tumor-necrosis-factor (TNF) therapy. Moreover, gut mesenchymal cells can increase their expression in HLA-DR in inflammatory conditions, acting as secondary antigen presenting cells [35]. Interestingly, we found an increase in HLA-DRA and HLA-DRB1 expression in the colon of NE patients. Thus, despite profound leuko-neutropenia, we hypothesize that NE may result from an unregulated inflammatory response mediated by IL-6 family cytokines. Although the transcriptomic analysis cannot distinguish inflammatory pathways activated in stromal cells versus epithelial cells or conventional immune cells, we hypothesize that stromal cells have the potential to assume the role of “conventional” immune cells and function as such in NE patients. However, we acknowledge that this study can only formulate hypotheses about the organization of the local inflammatory response. Further investigations, such as single-cell analyses, will be crucial to clarify these mechanisms.

Finally, the unsupervised analysis reveals a temporal shift from enterotype 2 to enterotypes 1 and 4 characterized by a substantial decrease in the relative abundance of butyrate-producing genera. This decrease correlates with reduced fecal butyrate concentrations and plasma citrulline levels. Butyrate, a SCFA produced by gut microbiota is crucial for maintaining the integrity of the enteric barrier. It is the preferential energy source used by colonocytes, increasing oxygen consumption and contributing to the maintenance of the gut’s anaerobic environment [36]. It also regulates the formation of tight junctions between intestinal epithelial cells [37], enhances mucus production [38], and stimulates the secretion of various antimicrobial substances by enterocytes while reducing the secretion of proinflammatory cytokines [39]. Tan *and colleagues* reported a decrease in OSM secretion in the presence of *Roseburia intestinalis*, a bacteria known for producing butyrate [40]. Hence, in NE patients, the depletion of butyrate production by the gut microbiota may have contributed to the up-regulation of the OSM-OSMR axis and enterocyte barrier dysfunction. Although a suitable animal model of NE has yet to be developed, animal studies would be valuable to address this question.

## Conclusions

In conclusion, our findings suggest that NE is a component of a broader spectrum of post-chemotherapy gastrointestinal damages characterized by a significant gut dysbiosis. Despite leukopenia, NE is associated with a local immune activation via the IL-6 and the OSM-OSMR pathways, which is not driven by neutrophils. The distinctive microbiological profile associated with NE in the gut microbiota features an overrepresentation of genera such as *Veillonella* genus, *or Enterococcus faecalis*, alongside a depletion of butyrate-producing genera that could contribute to perpetuating local inflammation. These findings open new avenues for future research into the role of OSM-OSMR in chemotherapy-related gastrointestinal damages and for nutritional management aimed at preserving SCFAs production.

## Electronic supplementary material

Below is the link to the electronic supplementary material.

Supplementary Material 1

## Data Availability

Data have been deposited to the National Center for Biotechnology Information Sequence Read Archive (Bioproject ID PRJNA1027838).

## Abbreviations

AML: Acute myeloid leukemia
BMI: Body mass index
FDR: False discovery rate
FLT3-inhibitor: Fms tyrosin kinase inhibitor
GvHD: Graft vs. Host disease
IBD: Inflammatory bowel diseases
ICU: Intensive care unit
i-FABP: Intestinal fatty-acid binding protein
IL: Interleukin
KEGG: Kyoto Encyclopedia of Genes and Genomes
L_2_FC: Log_2_ fold change
LIF: Leukemia inhibitor factor
NE: Neutropenic enterocolitis
PERMANOVA: Permutational analysis of Variance
SAPSII: Simplified Acute Physiology Score
SCFAs: Short chain fatty acids
SOFA: Sequential Organ Failure Assessment
STAT1: Signal transducer and activator of transcription 1
OSM: Oncostatin M
OSMR: Oncostatin M receptor
OTUs: Operational taxonomic units
TNF: Tumor necrosis factor
