# Supplementary material for: Unravelling neutropenic enterocolitis: insights from gut microbiota, and intestinal barrier analyses
Source: Exp Hematol Oncol. 2025 May 16;14:74. doi: 10.1186/s40164-025-00661-4 (PMC12084932; doi:10.1186/s40164-025-00661-4)
Supplement: Supplementary file 1 — Supplementary Material 1 [file 40164_2025_661_MOESM1_ESM.docx]

# Supplemental data

Natacha Kapandji^1,2,3^, Maud Salmona^4,5^, Anaïs Lemoine^2,3^, Guillaume Ulmann^6^, Julien Calderaro^7^, Brigitte Roche^8^, Nathalie Kapel^9,3^, Lucie Biard^10^, Etienne Lengline^11^, Jérôme Le Goff^4^, Christophe Rodriguez^12^, Muriel Thomas^2,3^, Lara Zafrani^1,13^

^1.^ Intensive Care Unit, Saint Louis Academic Hospital, AP-HP, Paris, France

^2.^ UMR1319, Micalis Institute, INRAe, AgroParisTech, Université Paris-Saclay, Jouy-en-Josas, France

^3.^ Paris Center for Microbiome Medicine (PaCeMM) FHU, AP-HP, Paris, France

^4.^ Virology department, Saint Louis Academic Hospital, AP-HP, Paris, France

^5.^ Team Insight, INSERM U976, Université Paris-Cité, Paris, France

^6.^ Clinical Chemistry Department, Cochin Academic Hospital, AP-HP, Paris, France

^7.^ Pathology department, Henri Mondor Academic Hospital, Créteil, AP-HP, France

^8.^ Pathology department, Saint Louis Academic Hospital, Paris, AP-HP, France

^9.^ Functional Coprology Laboratory, Pitié Salpêtrière Academic Hospital, AP-HP, Paris, France

^10.^ Clinical Trial Unit, Saint Louis Academic Hospital, AP-HP, Paris, France

^11.^ Hematology department, Saint Louis Academic Hospital, Paris, AP-HP, France

^12.^ Department of Microbiology, Henri Mondor Academic Hospital, Créteil, AP-HP, France

^13.^ INSERM UMR 944, Université Paris-Cité, Paris, France

**Corresponding author :** Dr Natacha Kapandji,

Intensive care unit, Saint Louis Academic Hospital, AP-HP, 1 avenue Claude Vellefaux 75010 Paris, France. Mail : [natacha.kapandji@aphp.fr](mailto:natacha.kapandji@aphp.fr) Phone 0033 6 67 33 92 14

## Supplemental Methods

### Patient recruitment, sample collection and clinical data and samples collection

Patients admitted to Saint-Louis University Center (Paris, France) for AML between September 1, 2020 and August 28, 2022 were prospectively recruited in the AML cohort. Patients with promyelocytic AML were excluded. They received chemotherapy and broad-spectrum antibiotics in case of neutropenic fever or septic complication at the discretion of the clinician. During neutropenia, patients presenting the 3 major criteria defined by Nesher and Rolston and no alternative diagnosis were diagnosed with NE and formed the AML-NE group. The remaining patients constituted the AML-control group, which was further divided into two subgroups: the AML-control diarrhea (+) group, comprising patients with uncomplicated diarrhea not related to NE, and the AML-control diarrhea (-) group, consisting of asymptomatic patients. Additionally, patients admitted to the ICU for NE with other underlying hematological diseases than AML formed the N-AML-NE group. The study was approved by the ethics committee “Comité de Protection des Personnes Ile de France VII” (N° ID-RCB : 2019-A02172-55). It was registered in Clinicaltrials.gov (identifier: NCT04438278). Reporting follow the STORMS (Strengthening The Organizing and Reporting of Microbiome Studies) guidelines (1).

### Clinical data collection

Clinical and microbiological data were collected from medical charts including age, gender, comorbidities, malnutrition at admission and discharge defined according to the French recommendations by the association of one etiological criteria (an evolutive cancer) and one phenotypic criteria among: weight loss ≥ 5% in 1 month or ≥ 10% in 6 months or ≥ 10% compared to the usual weight before the onset of the disease; Body mass index (BMI) < 18.5 kg/m2; quantified reduction in muscle mass and/or function (2), AML type based on the revised 4^th^ edition of the World Health Organization Classification (3), administered chemotherapy, and other received treatments notably antibiotics. In hospital complications, organ dysfunctions assessed by the Sequential Organ Failure Assessment (SOFA) score, ICU admission and length of stay as well as hospital length of stay, 90-days and 1-year outcomes were collected. In ICU, severity was evaluated by the Simplified Acute Physiology Score (SAPS II), which provides an estimate of the risk of death at admission (4).

### Fecal and plasma samples collection

Serial stool and blood samples were collected from the AML patients at 3 distinct time points: before the initiation of induction chemotherapy defining the all-AML baseline samples, on Day 14 which aligns with the median onset of NE as reported in existing literature (5), and after neutropenia recovery. Additional samples were obtained in case of uncomplicated diarrhea defining the AML-controls with diarrhea group (AML-C diarrhea (+)) or when a definitive diagnosis of NE was established defining the AML-NE diagnosis group. In the N-AML-NE cohort, samples were collected at the time of NE’s diagnosis. The stool samples were sent to the laboratory, aliquoted, and frozen at -80°C within 4 hours of collection.

***Fecal samples processing***

### Fecal samples processing for the PCR

Patients’ fecal frozen-aliquoted samples of 250 mg, were homogenized and lysed using both mechanical (bead beating for 10 minutes) and chemical techniques as previously described (6). Total DNA was extracted using the QIAamp PowerFecal DNA extraction kit (Qiagen®, Hilden, Germany), following the manufacturer's instructions.

### Quantitative PCR for bacterial load evaluation

Ten microliters of extracted DNA diluted at (1/1000) were added with 12.5µL PCR Buffer, 0.875 µL distilled-deionized water, 0.625 µL TaqMan™ MGB Probe (TM 1389F) (ThermoFisher®, Waltham, Massachusetts) and 0.5 µL of each TaqMan™ Primer (Bact 1369 and Prok1492) (ThermoFisher®, Waltham, Massachusetts). PCR conditions were 10 min at 95°C, followed by 40 cycles of 15 s at 95°C, and 1min at 60°C. The assays were performed using a StepOne™ Real-Time PCR System (ThermoFisher®, Waltham, Massachusetts). The standard curve was constructed using serial dilutions of *E. coli* S123 (7). Each sample was tested twice, and results were analyzed using StepOne™ Software v2.3 (ThermoFisher®, Waltham, Massachusetts).

### 16s rRNA gene sequencing and identification pipeline

Amplification of the V3-V4 region of the 16S rRNA gene and library preparation were carried out as outlined in the Illumina protocol for 16S sequencing, employing the primers S-D-Bact-0341-b-S-17 and S-D-Bact-0785-a-A-21 (8). The libraries underwent sequencing on the Illumina MiSeq platform (Illumina®, San Diego, California) using the MiSeq Reagent kit V3. Subsequently, raw sequences were analyzed utilizing the FROGS pipeline on the Galaxy Migale INRAe platform (9). Briefly, after demultiplexing the barcoded Illumina paired-end reads, sequences were cleaned, chimeras were removed, and operational taxonomic units (OTUs) were generated through single-linkage clustering. Taxonomic assignment was performed to determine the community composition using the RDP naïve Bayesian classifier (10). Sequence data were deposited in the National Center for Biotechnology Information Sequence Read Archive (Bioproject ID PRJNA1027838).

### Fecal Short chain fatty acids quantification

The quantification of SCFAs (acetate, propionate, butyrate, valerate, isobutyrate, isovalerate, caproate, and isocaproate) was conducted using the Gas-chromatograph 7890B (Agilent®, Santa-Clara, CA, USA) following the protocol previously described (11). Fragments weighing 100 to 250 mg from each stool sample were homogenized and diluted with distilled-deionized water in a 1:1 or 1:2 ratio depending on the sample’s consistency. After agitation for 2 hours at 4°C, the samples were centrifuged (12,000 g for 15 minutes at 4°C) and supernatant was collected. Proteins were precipitated using a phosphotungstic acid saturated solution overnight. Samples were centrifuged again (12,000 g for 15 minutes at 4°C) and supernatant collected. For each sample, a volume of 40 µL of the supernatant was used for the analyze to which 10 µL of 2-Ethylbutyric Acid 99% (ref 109959, Sigma-Aldrich®, Saint-Louis, Missouri, USA) was added as an internal control. A standard solution was prepared on the day of analysis with the Volatile Free Acid Mix (ref CRM46975, Sigma-Aldrich®, Saint-Louis, Missouri, USA) diluted in 40 µL of water and added with the internal control. All samples were analyzed in duplicate. The data was collected, and peaks integrated using OpenLab Chemstation (Agilent®, Santa-Clara, CA, USA). Results were expressed in micromolar units per gramme of dry weight sample.

*Fecal markers of inflammation dosages*

Fecal calprotectin (f-calprotectin), a protein secreted by neutrophils in the inflamed gut and currently used to monitor inflammatory bowel diseases, was used as a marker of neutrophil activation in the gut. Its levels were measured using a chemiluminescent immunoassay on an automated system (Liaison® XL, DiaSorin). The detection limit was 5 μg/g of feces. Human 𝛽-defensin 2 (f-hBD2) is an antimicrobial peptide secreted by the gastrointestinal epithelium in response to inflammation or infection. It was quantified in the feces by ELISA (Immundiagnostik® AG, Germany) according to manufacturer instructions. Absorbance measurements were performed on a BioTek Epoch2 microplate reader (Agilent®) and data analyzed using the BioTek Gen5 software (Agilent®). The quantification threshold was 0,2 ng/g of feces.

***Plasma samples processing***

### Plasma biosensors of enteric trophicity

Plasmatic citrulline concentration was measured by ion-exchange high performance liquid chromatography (Aminotac Amino Acid Analyzer, Jéol®, Croissy-sur-Seine, France). The vast majority of produced citrulline is taken up in the proximal convoluted tubules of the kidneys as a precursor for de novo arginine synthesis. Therefore, renal failure is associated with higher circulating citrulline levels especially when creatinine clearance is lower than 50 mL.min^-1^ (12). Hence, the measured concentrations were normalized to a creatinine clearance of 60 mL/min. The clearance used was calculated based on the day's creatinine measurement using the Cockcroft and Gault formula (13).

*Plasma cytokine and chemokines dosages*

Cytokines and chemokines concentrations were determined using Meso Scale Discovery (MSD) electrochemiluminescence method with the MESO QuickPlex SQ120 (MSD®, Rockville, Maryland, USA) device and the V-PLEX Proinflammatory Panel 1 (human) kit.

### Transcriptomic analysis from anatomical specimens

When NE’s patients underwent surgical management involving digestive resection, macroscopic and microscopic analysis were conducted by the pathology department in accordance with local practices. Paraffin-embedded fragments were collected for transcriptomic analysis. They were compared to samples taken from anatomical specimens of colectomies performed for colic tumors. Samples were at a distance from the tumor site and considered healthy. All samples underwent pre-extraction consisting of triple lysis: (i) mechanical with a mixture of glass beads of different diameters, (ii) chemical with the addition of 400µL RLT Plus Buffer (Qiagen®, Hilden, Germany) and (iii) enzymatic with the addition of Proteinase K (Qiagen®, Hilden, Germany) and incubation under agitation at 56°C for 1 hour. The lysate was then extracted using the DNA Midi Prep kit (Qiagen®, Hilden, Germany) on the Qiasymphony automated system (Qiagen®, Hilden, Germany) according to the manufacturer's instructions. RNA libraries were built from the extract using the TruSeq Stranded Total RNA (Illumina®, San Diego, California) kits according to the manufacturer's instructions and pair-end sequenced (2*150bp) using NovaSeq 6000 SP Reagent Kit v1.5 (300 cycles) on NovaSeq6000 (Illumina®, San Diego, California). The data analysis used several software performing the steps described below. First, the reads underwent quality filtering using Trimmomatic v0.39 (RRID:SCR_011848) followed by alignment and annotation on the human genome (Homo_sapiens. GRCh38) using hisat2v2.2.1 software. Samples with less than 10 million reads at the end of this step were excluded as well as those with less than 10,000 listed genes (excluding ribosomal genes, excluded from the analysis).

### Statistical analysis

*General analysis*

For univariate analysis, categorical variables expressed as numbers and percentage, were compared using χ^2^ test or Fisher's exact test (n < 5). Continuous variables expressed as mean (Standard Deviation) or median [interquartile range] according to their distribution tested by the Shapiro-Wilk test were compared using when 2 groups were compared Student's T-test for unpaired data, or Mann-Whitney U-Test when Levene's Test excluded homogeneity of variances). Kruskal-Wallis Test with Dunn’s multiple comparisons tests for more than 2 groups. Comparisons of matched data sets were performed using Friedman tests. Survival plots were drawn to evaluate 1-year outcome, and Log-Rank test was used for survival comparisons. ROC curve to evaluate the sensitivity and specificity of plasma citrulline dosage to detect NE was drawn and confidence interval was calculated using Wilson/Brown method. Correlations were tested using Spearman’s correlation coefficients. Missing data were not imputed. All comparisons were bilateral and a p-value < 0.05 was considered statistically significant. Statistical analyses were carried out using Jamovi software version 2.3.21.0 (RRID:SCR_016142) (63) and GraphPad software version 2.1.1 (RRID:SCR_002798) (223) for MacOS, Boston, Massachusetts USA, [www.graphpad.com](http://www.graphpad.com).

*Microbiome analysis*

Alpha and Beta Diversity analyses were conducted using the Phyloseq (RRID:SCR_013080) (16) and Vegan (RRID:SCR_011950) (17) packages in R. For Alpha diversity, the Simpson, Shannon, and Richness indices were calculated. For Beta diversity, Bray Curtis dissimilarity was employed. Principal Coordinate Analysis (PCoA) was performed using Bray Curtis. Permutational Analysis of Variance (PERMANOVA) was utilized to compare microbial communities between each group based on Bray Curtis dissimilarity indices, employing the adonis2 function within the R package Vegan. Sample clustering for enterotype assignment was achieved using hierarchical k-means with the 'factoextra' package (18). The DESeq2 package (RRID:SCR_000154) (21) was applied to identify bacteria with significant differential abundance at the species and genus levels among the different enterotypes. Differential abundance measurements were considered statistically significant if the adjusted p-value was < 0.01. The "coda4microbiome » R-package was used to identify meta-variables that would recapitulate a dynamic microbiome signature (19).

*Transcriptome analysis*

#### DeSeq2v1.30.1 software (RRID:SCR_000154) was used to normalize counting of the genes. Then, R software was used to perform the ontological analysis using FGSEA v1.16.0 package (RRID:SCR_020938) with the GSEA v7.5.1 databases (RRID:SCR_016863), the diagrams of Kyoto Encyclopedia of Genes and Genomes (KEGG) (RRID:SCR_012773) signaling pathways using Pathview v1.38.0 (RRID:SCR_002732) and heatmaps using pheatmap v1.0.12 (RRID:SCR_016418). All the metatranscriptomic process has been described in depth in a previous publication by Rodriguez and coworkers (20) .

## Supplemental Tables

**Supplemental Table S1 :**

Distribution of microorganisms identified in bloodstream infections among all patients.

**Supplemental Table S2 :**

Clinical characteristics of the AML and the N-AML-NE’s groups. AML: Acute myeloid Leukemia; ICU: Intensive Care Unit; NE: Neutropenic Enterocolitis; RRT: Renal Replacement Therapy; SAPS II: Simplified Acute Physiology Score; SOFA score: Sequential Organ Failure Assessment

**Supplemental Table S3 :**

Clinical characteristics of the 5 patients for whom a transcriptomic analysis was conducted. AML: Acute Myeloid Leukemia; DIC: Diffuse intravascular coagulation; DLBCL: Diffuse large B-cell lymphoma; F: Female; M: Male; NE: Neutropenic enterocolitis; PBSC: peripheral blood stem cell

**Supplemental Table S4 :**

Propionate and butyrate producing genera listed by Singh *and coworkers* and Reichardt *and coworkers* (21,22). * : Butyrate producing genera.

**Supplemental Table S1**

| **AML-cohort** | | | | **N-AML-NE** | | |
| --- | --- | --- | --- | --- | --- | --- |
| **AML-controls** | | **AML-NE** | |  |  |  |
|  | | *6 Enterobacteriaceae* | | *5 Enterobacteriaceae* | | |
|  | |  | *4 Escherichia coli* |  | *3 Escherichia coli* | |
|  | |  | *1 Klebsiella pneumoniae* |  | *1 Klebsiella pneumoniae* | |
|  | |  | *1 Enterobacter cloacae* |  | *1 Moraxella osloensis* | |
| *1 Enterococcus sp.* | | *4 Enterococcus sp.* | | *1 Enterococcus sp.* | | |
|  | *1 Enterococcus faecium* |  | *1 Enterococcus faecium* |  | *1 Enterococcus faecium* | |
|  |  |  | *3 Enterococcus faecalis* |  | |  |
| *2 Anaerobic bacteria* | | *3 Anaerobic bacteria* | |  | | |
|  | *1 Clostridium tertium* |  | *2 Clostridium tertium* |  | | |
|  | *1 Capnocytophaga gingivalis* |  | *1 Bacteroides fragilis* |  | | |
| *1 Candida sp.* | | *2 Candida sp.* | | *1 Candida sp.* | | |
|  | *1 Candida albicans* |  | *1 Candida albicans* |  | *1 Candida albicans* | |
|  |  |  | *1 Candida tropicalis* |  | | |
| *8 Staphylococcus sp.* | | *4 Staphylococcus sp.* | | *3 Staphylococcus sp.* | | |
|  | *5 S. haemolyticus* |  | *4 S. haemolyticus* |  | *2 S. haemolyticus* | |
|  | *3 S. epidermidis* |  |  |  | *1 S. epidermidis* | |
| *1 Pseudomonas aeruginosa* | | *1 Pseudomonas aeruginosa* | |  | | |
|  | | *1 Streptococcus oralis* | |  | | |

**Supplemental Table S2**

|  | **Neutropenic enterocolitis (n = 39)** | | | ***p*** |
| --- | --- | --- | --- | --- |
|  | **AML-NE (n = 26)** | | **N-AML-NE (n = 13)** |  |
| Age (years) [IQR] | 52 ± 15 | | |  |
|  | 52 ± 13 | | 51 ± 18 | 1.00 |
| Male – n (%) | 27 (69) | | |  |
|  | 19 (73) | | 8 (62) | 0.46 |
| Characteristics | | | | |
| Small bowel localization – n (%) | 20 (53) | | |  |
|  | 11 (44) | | 9 (69) | 0.14 |
| Caecum localization – n (%) | 3 (8) | | |  |
|  | 3 (12) | | 0 | 0.54 |
| Colon localization – n (%) | 29 (76) | | |  |
|  | 19 (76) | | 10 (77) | 0.95 |
| Time to NE’s diagnosis (days) [IQR] | 11 [9 – 14] | | |  |
|  | 11 [8 – 14] | | 11 [10 – 13] | 0.84 |
| SOFA score at NE’s diagnosis [IQR] | 6 [4 – 10] | | |  |
|  | 5 [4 – 8] | | 7 [6 – 10] | 0.13 |
| SAPS II of NE’s episode [IQR] | 54 [45 – 66] | | |  |
|  | 58 [48 – 69] | | 50 [45 – 63] | 0.43 |
| Complications | | | | |
| Bloodstream infections – n | 23 | | |  |
|  | 15 | | 8 | 0.83 |
| Local complications – n (%) | 4 (10) | | |  |
| Occlusive syndrome | 1 (4) | | 0 | 0.51 |
| Gastrointestinal bleeding | 1 (4) | | 2 (15) |  |
| Management | | | | |
| Surgery – n (%) | 10 (26) | | |  |
|  | 7 (27) | | 3 (23) | 1.00 |
| Digestive resection – n (%) | 6 (15) | | |  |
|  | 5 (19) | | 1 (7) | 0.64 |
| Mechanical ventilation – n (%) | 14 (36) | | |  |
|  | 9 (35) | | 5 (39) | 1.00 |
| Vasopressor support – n (%) | 10 (51) | | |  |
|  | 10 (39) | | 10 (77) | 0.02 |
| RRT – n (%) | 8 (21) | | |  |
|  | 6 (23) | | 2 (15) | 0.69 |
| Outcome | | | | |
| ICU length of stay (days) [IQR] | 9 [6 – 21] | | |  |
|  | 9 [6 – 26] | | 6 [4 – 15] | 0.13 |
| Hospital length of stay (days) [IQR] | 37 [29 – 51] | | |  |
|  | 33 [29 – 46] | | 46 [37 – 54] | 0.72 |
| In Hospital mortality rate – n (%) | 7 (18) | | |  |
|  | 5 (19) | | 2 (15) | 1.00 |
| Day 90 mortality rate – n (%) | 9 (23) | | |  |
|  | 6 (23) | 3 (23) | | 1.00 |

**Supplemental Table S3**

| **Age / Sexe** | **Comorbidities** | **Hematological diagnosis** | **Symptoms before chemotherapy** | **Treatment** | **Radiological localization** | **Identified Bloodstream microorganism** | **SAPS II / admission SOFA score** | **Ventilation duration (days)** | **Vasopressor support (days)** | **Renal replace-ment therapy (days)** | **Outcome** |
| --- | --- | --- | --- | --- | --- | --- | --- | --- | --- | --- | --- |
| 62/ M | - | DLBCL | - | Autologous PBSC supporting high-dose chemotherapy | Enterocolitis with digestive hemorrhage | - | 67 / 12 | 19 | 19 | 3 | Died in hospital at Day 54 |
| 55/ M | - | AML | Abdominal pain | Aracytine and Anthracycline    Anti-CD33 antibody | Colitis | - | 53 / 5 | 14 | 3 | 12 | Discharged at Day 68 alive and in remission |
| 52 / F | - | AML | - | Aracytine and Anthracycline  FLT3 inhibitors | Enterocolitis | *Escherichia coli*  *Enterococcus faecalis* | 67 / 11 | 10 | 2 | 4 | Discharged at Day 64 alive and in remission |
| 54 / F | Diabetes mellitus | AML with leukocytosis, DIC, leukostasis and tumor lysis syndrome | - | Aracytine and Anthracycline  FLT3 inhibitors  Hydroxyurea | Colitis | *Candida albicans* | 90 / 8 | 39 | 14 | 39 | Died in hospital at Day 52 |
| 59/ M | Chronic heart failure / Tabaco / Diabetes mellitus | AML | Diarrhea Abdominal pain Malnutrition | Aracytine and Anthracycline  Anti-CD33 antibody | Colitis | - | 75 / 10 | 13 | 5 | 0 | Discharged at Day 66 alive and in remission |

**Supplemental Table S4**

| **Class** | **Family** | **Genera** | **Species** |
| --- | --- | --- | --- |
| *Clostridia* | *Lachnospiraceae* | *Agathobacter** | *Eubacterium rectale** |
|  |  | *Roseburia** | *Roseburia inulinivorans** |
|  |  |  | *Roseburia intestinalis** |
|  |  |  | *Roseburia faecis** |
|  |  |  | *Roseburia hominis** |
|  |  | *Anaerobutyricum** | *Eubacterium hallii** |
|  |  | *Eubacterium** | *Eubacterium ramulus** |
|  |  |  | *Eubacterium limosum** |
|  |  | *Anaerostipes** | *Anaerostipes hadrus** |
|  |  |  | *Anaerostipes caccae** |
|  |  |  | *Anaerostipes butyraticus** |
|  |  |  | *Anaerostipes rhamnosivorans** |
|  |  | *Coprococcus** | *Coprococcus eutactus** |
|  |  |  | *Coprococcus comes** |
|  |  |  | *Coprococcus catus** |
|  |  | *Blautia* | *Blautia obeum* |
|  |  | *Anaerobutyricum** | *Anaerobutyricum soehngenii** |
|  |  | *Butyrivibrio** | *Butyrivibrio fibrisolvens** |
|  |  | *Mediterraneibacter* | *Ruminococcus gnavus* |
|  |  |  | *Ruminococcus torques* |
|  |  | *Lachnoclostridium* | *Clostridium symbiosum* |
|  |  | *Enterocloster* | *Clostridium asparagiforme* |
|  | *Oscillospiraceae* | *Faecalibacterium** | *Faecalibacterium prausnitzii** |
|  |  | *Subdoligranulum** | *Subdoligranulum variabile** |
|  |  | *Ruminococcus** | *Ruminococcus bromii* |
|  |  |  | *Ruminococcus callidus** |
|  |  |  | *Ruminococcus champanellensis** |
|  |  | *Anaerotruncus** | *Anaerotruncus colihominis** |
|  |  | *Incerteae sedis* | *Eubacterium spiraeum* |
|  | *Eubacteriales* | *Incerteae sedis* | *Clostridiales bacterium* |
| *Negativitutes* | *Veillonellaceae* | *Dialister* | *Dialister succinatiphilus* |
|  |  | *Veillonella* | *Veillonella parvula* |
|  | *Acidaminococacceae* | *Phascolarctobactereium* | *Phascolarctobacterium succinatutens* |
| *Verrucomicrobiae* | *Akkermansiaceae* | *Akkermensia* | *Akkermensia muciniphila* |
| *Actinomycetes* | *Bifidobacteriaceae* | *Bifidobacterium* | *Bifidobacterium adolescentis* |
| *Bacteroidia* | *Bacteroidaceae* | *Bacteroides* | *Bacteroides thetaiotamicron* |
|  |  |  | *Bacteroides vulgatus* |
|  |  |  | *Bacteroides fragilis* |

## Supplemental Figures

## Supplemental Figure S1: Distribution of antibiotics received before chemotherapy

## Histogram depicting the classes and durations of antibiotics received by each patient prior to induction chemotherapy. For two patients, the duration of Amoxicillin treatment was unknown (UV: unknown value).

## Supplemental Figure S2: Survival plot among the AML cohort

Survival Plot of AML-NE and AML-controls within the first year. *p-value* was calculated with a Log-rank Test. p*-value* < 0.01 was considered statistically significant.

**Supplemental Figure S3: Impact of Antibiotics on baseline microbiota’s characteristics**

(A) Violin plots illustrating bacterial load levels (expressed as number of bacteria / g of feces), and 𝛼-diversity indices (number of OTUs, Shannon and Simson indices) in baseline samples of patients who received antibiotics (ATB (+) , n = 25) or not (ATB (-) , n = 13). *p-values* were calculated using a non-parametric Mann-Whitney test for unpaired data. A *p-value* < 0.01 was considered statistically significant. (B) Bar plot showing the distribution of the 4 enterotypes at baseline between the 2 groups. *p-value* was calculated using a Fisher’s exact test with a threshold for significatively of 0.01.

**Supplemental Figure S4: Correlation between SCFA stool concentrations and SCFA-producers genera**

Spearman correlation and linear regression of (A) fecal concentration of butyrate (µmol/g of dry weight) with the number of OTUs of butyrate-producers’ genera and (B) fecal concentration of propionate (µmol/g of dry weight) with the number of OTUs of propionate-producers’ genera.

**Supplemental Figure S6 : Cell adhesion molecules KEGG pathway**

(A) Schematic presentation of the cell adhesion molecules KEGG pathway (hsa04514) focusing on immune cells interactions lead by class II major histocompatibility complex (colored in red). Mann-Whitney comparison between NE samples and controls of normalized expression count for (B) HLA-DRB1 gene, (C) HLA-DRA gene, (D) HLA-DQB1 gene, and (E) HLA-B gene. p-value < 0.05 was considered statistically significant.

**Supplemental Figure S1:**


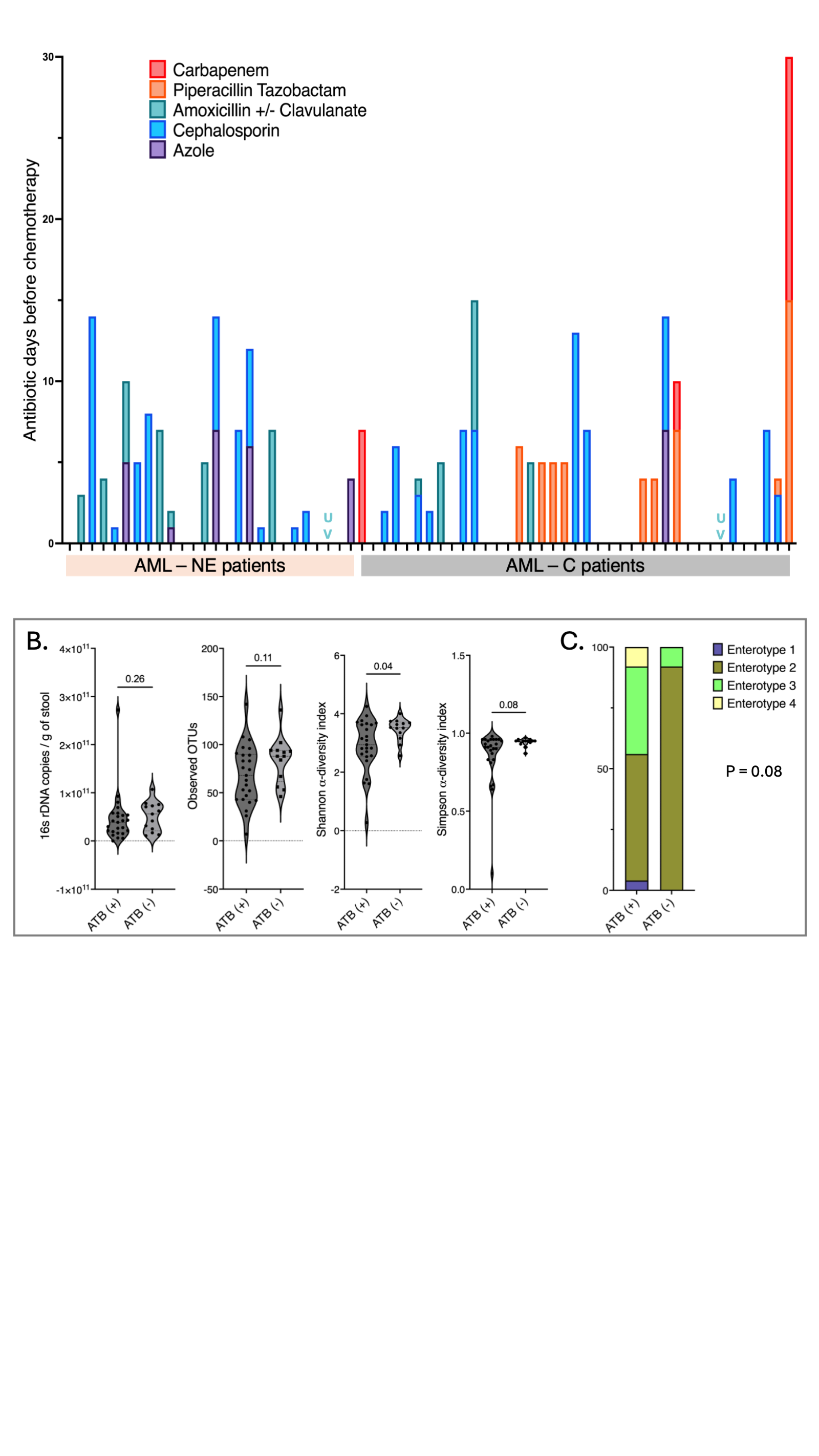


**Supplemental Figure S2:**

**
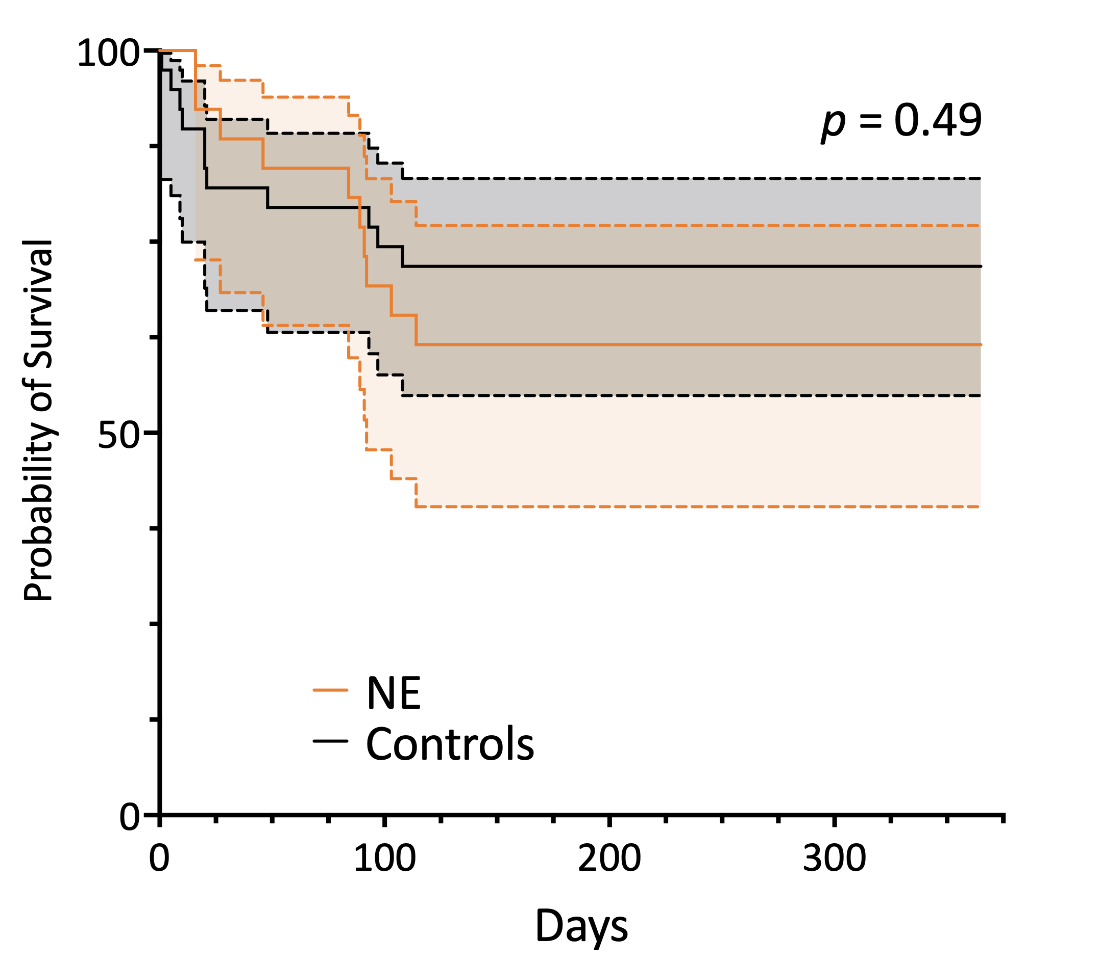
**

**Supplemental Figure S3:**

**
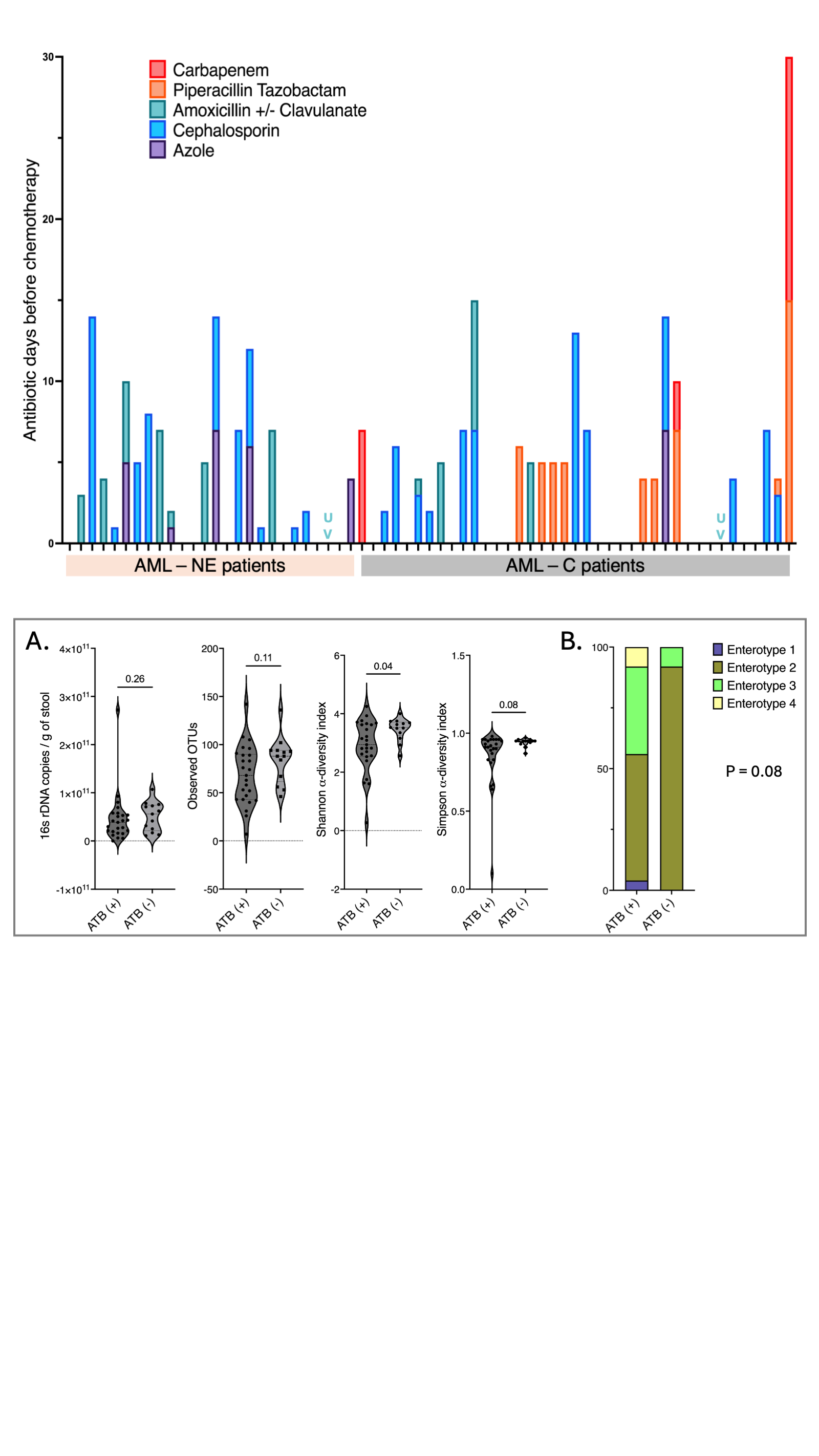
**

**
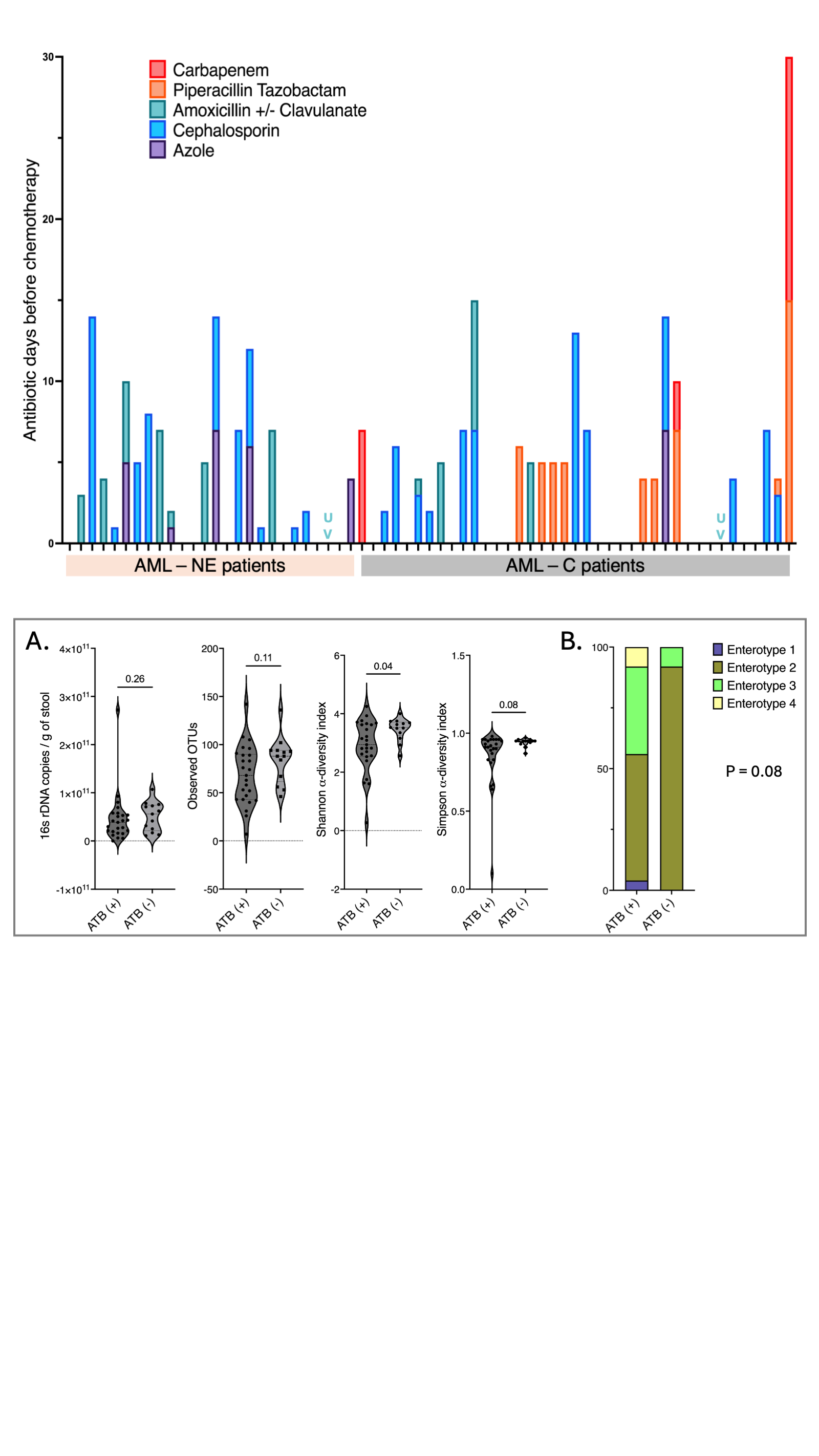
**

**Supplemental Figure S4:**

A.

B.

**Supplemental Figure S5**

## Supplemental references

1. Mirzayi C, Renson A, Zohra F, Elsafoury S, Geistlinger L, Kasselman LJ, et al. Reporting guidelines for human microbiome research: the STORMS checklist. Nat Med. nov 2021;27(11):1885‑92.

2. Alexandre P. Haute Autorité de santé. 2019;

3. Hwang SM. Classification of acute myeloid leukemia. Blood Res. 30 juill 2020;55(Suppl):S1‑4.

4. Le Gall JR, Lemeshow S, Saulnier F. A new Simplified Acute Physiology Score (SAPS II) based on a European/North American multicenter study. JAMA. 22 déc 1993;270(24):2957‑63.

5. Duceau B, Picard M, Pirracchio R, Wanquet A, Pène F, Merceron S, et al. Neutropenic Enterocolitis in Critically Ill Patients: Spectrum of the Disease and Risk of Invasive Fungal Disease. Crit Care Med. févr 2019;1.

6. Qin J, Li R, Raes J, Arumugam M, Burgdorf KS, Manichanh C, et al. A human gut microbial gene catalog established by metagenomic sequencing. Nature. 4 mars 2010;464(7285):59‑65.

7. Mayeur C, Gratadoux JJ, Bridonneau C, Chegdani F, Larroque B, Kapel N, et al. Faecal D/L lactate ratio is a metabolic signature of microbiota imbalance in patients with short bowel syndrome. PloS One. 2013;8(1):e54335.

8. Klindworth A, Pruesse E, Schweer T, Peplies J, Quast C, Horn M, et al. Evaluation of general 16S ribosomal RNA gene PCR primers for classical and next-generation sequencing-based diversity studies. Nucleic Acids Res. 7 janv 2013;41(1):e1.

9. Escudié F, Auer L, Bernard M, Mariadassou M, Cauquil L, Vidal K, et al. FROGS: Find, Rapidly, OTUs with Galaxy Solution. Bioinforma Oxf Engl. 15 avr 2018;34(8):1287‑94.

10. Cole JR, Wang Q, Cardenas E, Fish J, Chai B, Farris RJ, et al. The Ribosomal Database Project: improved alignments and new tools for rRNA analysis. Nucleic Acids Res. 1 janv 2009;37(suppl_1):D141‑5.

11. Cherbuy C, Bellet D, Robert V, Mayeur C, Schwiertz A, Langella P. Modulation of the Caecal Gut Microbiota of Mice by Dietary Supplement Containing Resistant Starch: Impact Is Donor-Dependent. Front Microbiol. 6 juin 2019;10:1234.

12. Pironi L, Guidetti M, Lauro A, Zanfi C, Agostini F, D’Errico A, et al. Plasma citrulline after small bowel transplantation: effect of time from transplantation, acute cellular rejection, and renal failure. Clin Transplant. nov 2015;29(11):1039‑46.

13. Cockcroft DW, Gault H. Prediction of Creatinine Clearance from Serum Creatinine. Nephron. 28 nov 2008;16(1):31‑41.

14. jamovi - open statistical software for the desktop and cloud [Internet]. [cité 5 sept 2023]. Disponible sur: https://www.jamovi.org/

15. The Comprehensive R Archive Network [Internet]. [cité 5 sept 2023]. Disponible sur: https://cran.r-project.org/

16. McMurdie PJ, Holmes S. phyloseq: An R Package for Reproducible Interactive Analysis and Graphics of Microbiome Census Data. PLOS ONE. 22 avr 2013;8(4):e61217.

17. Oksanen J, Simpson GL, Blanchet FG, Kindt R, Legendre P, Minchin PR, et al. vegan: Community Ecology Package [Internet]. 2022 [cité 30 août 2023]. Disponible sur: https://cran.r-project.org/web/packages/vegan/index.html

18. Kassambara A, Mundt F. factoextra: Extract and Visualize the Results of Multivariate Data Analyses [Internet]. 2020 [cité 30 août 2023]. Disponible sur: https://cran.r-project.org/web/packages/factoextra/index.html

19. Calle ML, Susin A. Identification of Dynamic Microbial Signatures in Longitudinal Studies [Internet]. bioRxiv; 2022 [cité 4 sept 2023]. p. 2022.04.25.489415. Disponible sur: https://www.biorxiv.org/content/10.1101/2022.04.25.489415v1

20. Rodriguez C, Prost N de, Fourati S, Lamoureux C, Gricourt G, N’debi M, et al. Viral genomic, metagenomic and human transcriptomic characterization and prediction of the clinical forms of COVID-19. PLOS Pathog. 29 mars 2021;17(3):e1009416.

21. Singh V, Lee G, Son H, Koh H, Kim ES, Unno T, et al. Butyrate producers, « The Sentinel of Gut »: Their intestinal significance with and beyond butyrate, and prospective use as microbial therapeutics. Front Microbiol. 2022;13:1103836.

22. Reichardt N, Duncan SH, Young P, Belenguer A, McWilliam Leitch C, Scott KP, et al. Phylogenetic distribution of three pathways for propionate production within the human gut microbiota. ISME J. juin 2014;8(6):1323‑35.
